# Sequestration by IFIT1 Impairs Translation of 2′O-unmethylated Capped RNA

**DOI:** 10.1371/journal.ppat.1003663

**Published:** 2013-10-03

**Authors:** Matthias Habjan, Philipp Hubel, Livia Lacerda, Christian Benda, Cathleen Holze, Christian H. Eberl, Angelika Mann, Eveline Kindler, Cristina Gil-Cruz, John Ziebuhr, Volker Thiel, Andreas Pichlmair

**Affiliations:** 1 Innate Immunity Laboratory, Max-Planck Institute of Biochemistry, Martinsried/Munich, Germany; 2 Department of Structural Cell Biology, Max-Planck Institute of Biochemistry, Martinsried/Munich, Germany; 3 Department of Proteomics and Signal Transduction, Max-Planck Institute of Biochemistry, Martinsried/Munich, Germany; 4 Institute of Immunobiology, Kantonspital St. Gallen, St. Gallen, Switzerland; 5 Institute of Medical Virology, Justus Liebig University, Giessen, Germany; 6 Vetsuisse Faculty, University of Zürich, Zürich, Switzerland; Washington University School of Medicine, United States of America

## Abstract

Viruses that generate capped RNA lacking 2′O methylation on the first ribose are severely affected by the antiviral activity of Type I interferons. We used proteome-wide affinity purification coupled to mass spectrometry to identify human and mouse proteins specifically binding to capped RNA with different methylation states. This analysis, complemented with functional validation experiments, revealed that IFIT1 is the sole interferon-induced protein displaying higher affinity for unmethylated than for methylated capped RNA. IFIT1 tethers a species-specific protein complex consisting of other IFITs to RNA. Pulsed stable isotope labelling with amino acids in cell culture coupled to mass spectrometry as well as *in vitro* competition assays indicate that IFIT1 sequesters 2′O-unmethylated capped RNA and thereby impairs binding of eukaryotic translation initiation factors to 2′O-unmethylated RNA template, which results in inhibition of translation. The specificity of IFIT1 for 2′O-unmethylated RNA serves as potent antiviral mechanism against viruses lacking 2′O-methyltransferase activity and at the same time allows unperturbed progression of the antiviral program in infected cells.

## Author Summary

Cellular messenger RNAs of higher eukaryotes are capped with a methylated guanine and, in addition, methylated at the 2′O position of the first ribose. Viruses unable to methylate their RNA at the 2′O position of the cap and viruses generating uncapped RNA with 5′ triphosphate groups are inhibited by an antiviral complex of different IFIT proteins. How IFIT proteins restrict viruses lacking 2′O methylation at the RNA cap remained unclear. We used a mass spectrometry-based approach to identify proteins binding to capped RNA with different methylation states. We found that IFIT1 directly binds to capped RNA and that this binding was dependent on the methylation state of the cap. Having identified IFIT1 as being central for recognition of 2′O-unmethylated viral RNA we further examined the mode of action of IFITs *in vitro* and *in vivo*. Our experiments clearly show that the antiviral mechanism of IFIT1 is based on sequestration of viral RNA lacking cap 2′O methylation, thereby selectively preventing translation of viral RNA. Our data establish IFIT1 as a general sensor for RNA 5′ end structures and provide an important missing link in our understanding of the antiviral activity of IFIT proteins.

## Introduction

Effective control of viral infection by host organisms requires sensing of pathogens and activation of appropriate defence mechanisms [1]–[3]. One component commonly sensed by the host is viral genetic material, whether DNA delivered to the cytoplasm through viral infection or viral RNA bearing motifs not commonly found on eukaryotic RNAs [4], [5]. Most cellular cytoplasmic RNAs are single-stranded, and bear a 5′monophosphate (rRNAs and tRNAs), or an N7 methylated guanosine cap (mRNAs) linked via a 5′-to-5′ triphosphate bridge to the first base. In higher eukaryotes, mRNA is further methylated at the 2′O position of the first ribose [6], [7]. Viruses, in contrast, can form long double-stranded RNA (dsRNA) and generate RNAs bearing 5′triphosphosphates (PPP-RNA) or RNAs lacking methylation [8]–[10]. All these distinct features of viral as opposed to cellular RNAs have been shown to activate the innate immune system and elicit synthesis of antiviral cytokines including Type I interferons (IFN-α/β), which ultimately restrict virus growth [11]–[14]. Among the proteins that sense viral RNA and are linked to IFN-α/β synthesis are retinoic acid-inducible gene I (RIG-I) and melanoma differentiation-associated gene 5 (Mda-5), which form the family of RIG-like receptors (RLRs) [5]. A further set of host proteins appears to bind virus-derived RNAs to directly inhibit virus production [8]. Several of these proteins are highly expressed upon stimulation of cells with cytokines like IFN-α/β and their antiviral effects become apparent only after binding to virus-derived nucleic acid. Prominent examples for such proteins are dsRNA binding proteins such as dsRNA-activated protein kinase R and 2′-5′ oligoadenylate synthetase, and proteins that bind PPP-RNA, like interferon-induced proteins with tetratricopeptide repeats (IFIT) 1 and -5 [3], [15], [16]. Little is known about the repertoire of cellular proteins that recognise unmethylated cap structures, although replication of viruses with inactive RNA 2′O methyltransferase is strongly inhibited by IFN-α/β *in vitro* and *in vivo* [11], [15]. Some of this antiviral activity has been genetically linked to Ifit1 and -2 in mice [17]–[19]. Here, we used an unbiased mass-spectrometry-based approach to identify cellular proteins that bind to 5′ unmethylated and methylated capped RNA, and explored their contribution to antiviral host responses.

## Results

### Identification of human and mouse proteins that bind capped RNA

**Figure 1:**
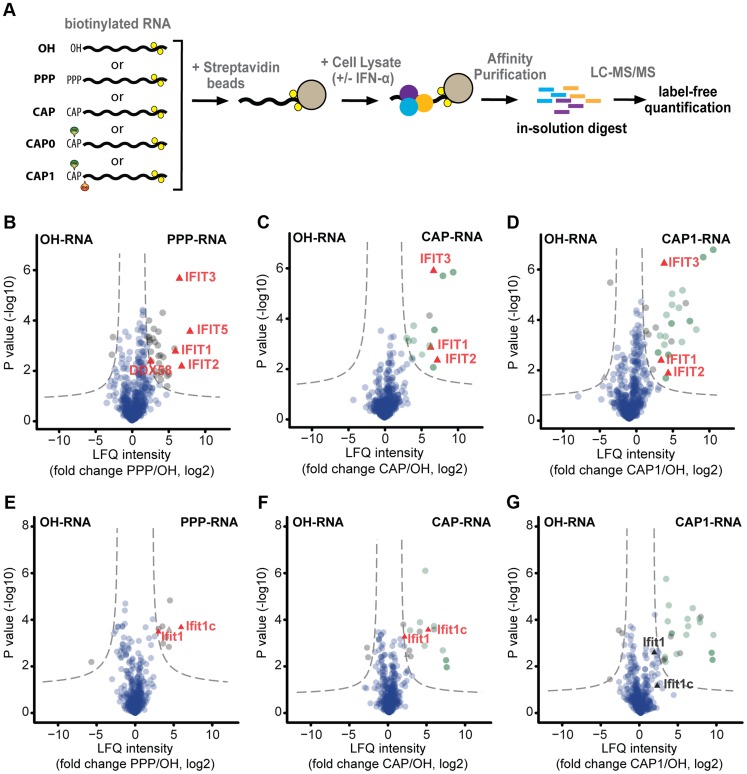
Mass spectrometry-based identification of human and murine interactors of capped RNA. (**a**) Schematic depiction of the experimental approach used for mass spectrometry (MS)-based identification of cellular RNA binding proteins. Biotinylated RNA with different 5′ end structures (OH, PPP, CAP, CAP0, CAP1) was coupled to streptavidin beads, and incubated with lysates obtained from cells that had been left untreated or treated with 1000 U/ml IFN-α for 16 h. Bound proteins were denatured, alkylated and directly digested with trypsin. The resulting peptides were subjected to shotgun liquid chromatography-tandem MS (LC-MS/MS). Three independent experiments were performed for each RNA bait, and the data were analysed with the MaxQuant software [37] using the label-free quantification algorithm [38]. (**b–d**) Proteins obtained from lysates of IFN-α-treated HeLa cells using the indicated biotinylated RNA baits were analysed by LC-MS/MS. Volcano plots show the degrees of enrichment (ratio of label-free quantitation (LFQ) protein intensities; x-axis) and p-values (t-test; y-axis) by PPP-RNA (**b**), CAP-RNA (**c**), and CAP1-RNA (**d**) baits as compared to OH-RNA. Significantly enriched interactors (see Materials and Methods) are separated by a hyperbolic curve (dotted line) from background proteins (blue dots), known cap-binding proteins (dark-green), and proteins known to associate with capped RNA (light green). Interferon-induced proteins [21] detected in the significantly enriched fractions (IFIT1-3 and 5, DDX58) are highlighted (red triangles). (**e–g**) As in (**b–d**) but for lysates of IFN-α-treated mouse embryo fibroblasts (MEFs). The interferon-induced proteins Ifit1 and Ifit1c [42] in significantly enriched and non-enriched fractions are highlighted.

To identify proteins that interact with 5′ capped RNA we used a proteomics approach based on affinity purification and mass spectrometry (AP-MS) [16]. RNA bearing terminal 5′ hydroxyl (OH-RNA), 5′ triphosphate (PPP-RNA), an unmethylated cap (CAP-RNA), a guanosine-N7 methylated cap (CAP0-RNA), or a guanosine-N7 methylated cap and a ribose-2′O methylated first nucleotide (CAP1-RNA) was coupled to agarose beads. The beads were then incubated with lysates of naïve HeLa cells or HeLa cells treated with IFN-α to increase the abundance of antiviral proteins (Fig. 1a, Fig. S1). By employing liquid-chromatography coupled to tandem mass spectrometry (LC-MS/MS) followed by quantitative interaction proteomics analysis, we identified 528 proteins that interacted with unmodified or RNA-coated beads (Fig. S2a, Table S1). While a large number of proteins were equally well represented in the bound fractions obtained with all RNAs (Fig. S2a), 68 proteins were found to be significantly enriched in samples recovered with 5′modified RNA compared to OH-RNA (Fig. S2b). As expected, the PPP-RNA binding proteins RIG-I (DDX-58), the IFIT1, -2, -3 complex and IFIT5 were enriched in PPP-RNA affinity purifications of IFN-α-treated HeLa cell lysate (Fig. 1b), validating the approach and confirming previous data [16]. Using unmethylated CAP-RNA as bait, we significantly enriched for proteins known to associate with cellular capped RNA (12 of 16 proteins) (Fig. 1c, Fig. S2b, Table S1). However, an important feature of cellular mRNAs is methylation on the N7 position of the guanosine cap and the ribose-2′O position of the first nucleotide (CAP1). N7 methylation is known to increase the affinity of the cap structure for proteins such as EIF4E and other cap-binding proteins [6], [7]. A methylation-dependent increase in protein binding was also evident in our AP-MS analysis when unmethylated CAP-RNA and methylated CAP1-RNA were used as baits (16 vs. 27 identified proteins), as the latter captured a higher number of significantly enriched proteins and, overall, these were enriched to a greater degree, as measured by label-free quantification (Fig. 1c–d, Table S1). Notably, we identified IFIT1, -2 and -3 among the uncharacterised CAP-RNA binding proteins, suggesting that the IFIT complex binds to RNA in a cap-dependent manner (Fig. 1c). IFIT5, which shows 57.2% aminoacid sequence identity and 75.6% similarity to IFIT1 and has recently been shown to form a tight binding pocket that specifically accommodates PPP-RNA [20], was not detected in fractions that bound capped RNA. When we compared our AP-MS dataset with transcriptome data of interferon-stimulated cells [21], IFITs were the only interferon-induced proteins found to be specifically enriched in CAP-RNA purifications, suggesting a predominant role of IFITs in innate immune responses directed against CAP-RNA (Fig. 1c, Fig. S2b). To analyse whether the set of proteins that binds to 5′modified RNA is conserved in other species, we performed the same AP-MS analysis on lysates of naïve and IFN-α-treated mouse embryonic fibroblasts (MEFs) (Fig. S3a, b, Table S2). Surprisingly, although PPP-RNA specifically enriched for Ifit1, the abundance of Ifit2 and Ifit3 was not increased (Fig. 1e). Instead we found enrichment of Ifit1c (also known as Gm14446), an uncharacterised IFIT protein that is strongly induced by IFN-α/β or virus infection (Fig. S4), suggesting that the architecture of the murine IFIT complex differs from that of its human counterpart. Significant enrichment for Ifit1 and Ifit1c could also be achieved with unmethylated CAP-RNA, but not with methylated CAP1-RNA, despite the fact that the latter bait captured more proteins with higher enrichment scores (Fig. 1f, g). We concluded from these analyses that, in both human and mouse, the IFIT complex is the only IFN-induced component that shows significant affinity for capped RNA.

### IFIT1 is the only IFIT that binds capped RNA

**Figure 2:**
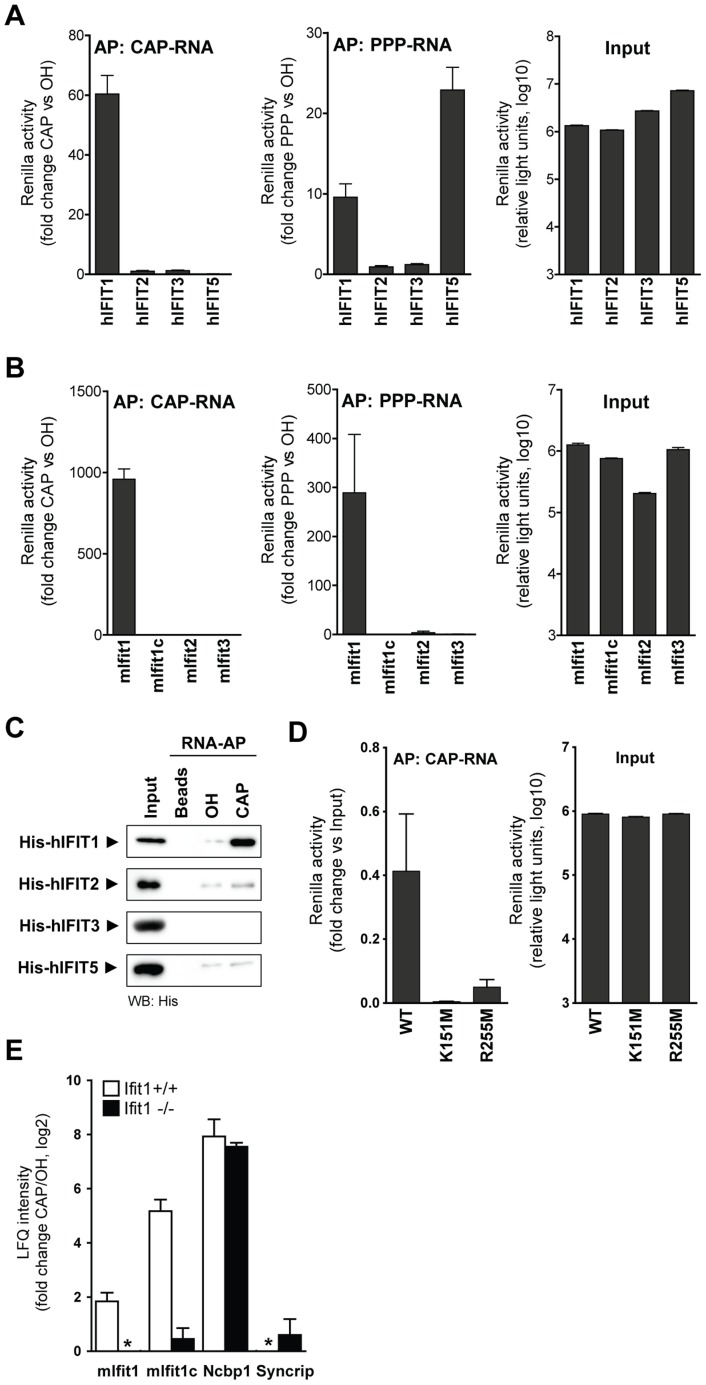
Human and mouse IFIT1 bind directly to unmethylated capped RNA. (**a**) Isolation of luciferase-tagged human IFIT (hIFIT) proteins from transfected 293T cells with beads coated with 250 ng RNA bearing 5′ OH, PPP or CAP. The graphs show luciferase activity after affinity purification (AP) with PPP-RNA and CAP-RNA (normalized to OH-RNA) and the activity of 10% of the input lysates. (**b**) Data obtained (as in **a**) for luciferase-tagged murine Ifit (mIfit) proteins affinity purified with PPP-RNA and CAP-RNA. (**c**) Recombinant His-tagged hIFIT1, -2, -3, and -5 were incubated with beads only or beads coated with OH-RNA or CAP-RNA. Bound proteins were detected by western blotting. Input shows 1/10^th^ of the amount incubated with beads. (**d**) Purification of luciferase-tagged wild-type (WT) and hIFIT1 mutants with CAP-RNA-coated beads. The graphs show luciferase activity after affinity purification and the activity of 10% of the input lysates. (**e**) Ratios of LFQ intensities of proteins identified by mass spectrometry in precipitates of CAP-RNA vs. OH-RNA in IFN-α-treated MEFs from wild-type (Ifit1^+/+^, grey bars) and Ifit1-deficient (Ifit1^−/−^, black bars) C57BL/6 mice. Error bars indicate means (±SD) from three independent affinity purifications. Asterisks indicate ratios with negative values.

Since human IFIT1, -2 and -3 associate with each other to form a multiprotein complex, we wished to determine which of them was responsible for tethering the IFIT complex to unmethylated CAP-RNA. We overexpressed each of the IFIT proteins, tagged with *Renilla* luciferase, in 293T cells and performed affinity purifications using OH-RNA, PPP-RNA and CAP-RNA. Remarkably, only human and murine IFIT1 were detected when CAP-RNA was used as bait (Fig. 2a, b), suggesting that IFIT1 mediates binding of the IFIT complex to CAP-RNA. Consistent with the MS analysis, IFIT5 exclusively bound to PPP-RNA but not to CAP-RNA. To exclude contribution of cellular factors to the interaction between IFIT1 and CAP-RNA we used recombinant human IFIT proteins for RNA precipitations which confirmed a direct interaction of IFIT1 with capped RNA (Fig. 2c). A structure-based modelling approach using IFIT5 [20] as template suggested that the RNA-binding cavity of IFIT1 is ∼700 Å^3^ larger than that of IFIT5 (Fig. S5) – implying that IFIT1 has slightly different RNA-binding properties. However, a lysine at position 151 and an arginine at position 255 of IFIT1, two residues involved in binding the terminal 5′ triphosphate group on PPP-RNA by IFIT5 and IFIT1 [20], were also required for binding of IFIT1 to capped RNA (Fig. 2d), indicating an overall similar mode of binding.

To provide additional evidence that binding of IFIT1 is indeed responsible for associating the IFIT complex to CAP-RNA, we performed AP-MS experiments on wild-type (Ifit1^+/+^) and mutant, Ifit1-deficient (Ifit1^−/−^) MEFs. The overall precipitation efficiency was comparable in both cell types, as evidenced by equal enrichment of the RNA-binding protein Syncrip and the cap-binding protein Ncbp1 (Fig. 2e and Fig. S4b). Ifit1c was not enriched in precipitates from Ifit1^−/−^ MEFs, which is consistent with the notion that the murine Ifit complex binds to CAP-RNA through Ifit1. These results show that the specific binding properties of IFIT1 are essential for recruitment of the human and murine IFIT complexes to their RNA targets.

### IFIT1 binding depends on the methylation status of the RNA cap

**Figure 3:**
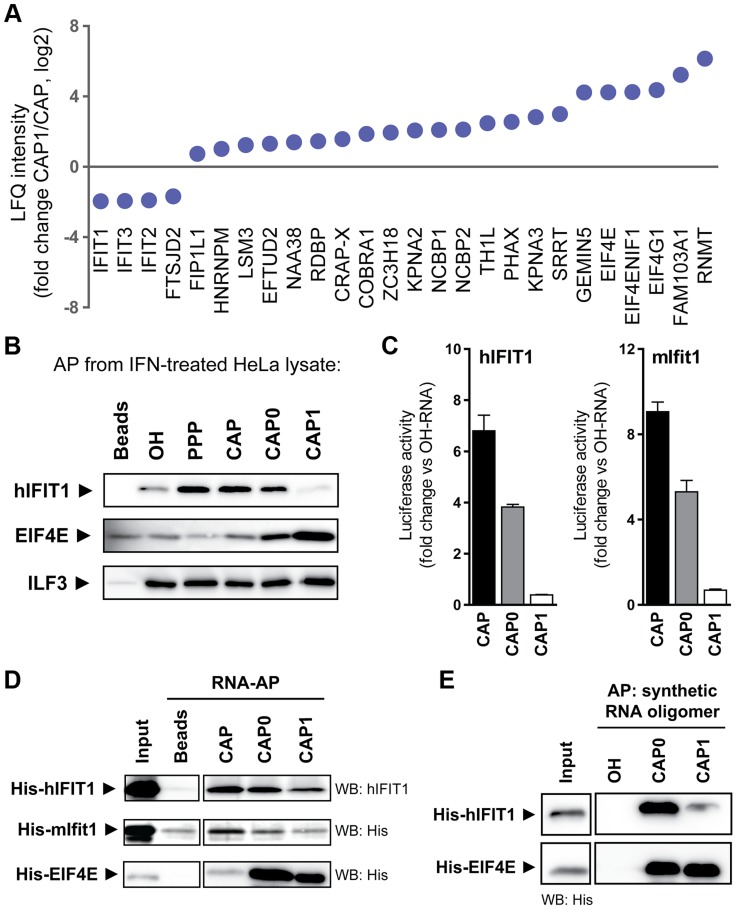
IFIT1 binds capped RNA in a methylation state-dependent manner. (**a**) Ratio of LFQ intensities of proteins identified by LC-MS/MS as significantly enriched in CAP1-RNA relative to CAP-RNA affinity purifications from IFN-treated HeLa cells, after filtering against the set of proteins that showed enrichment relative to 5′ OH-RNA(see Fig. S2b). Error bars indicate means (±SD) from three independent affinity purifications. (**b**) Precipitation of endogenous proteins from lysates of IFN-α treated HeLa cells with biotinylated RNA bearing 5′ OH, PPP, CAP, CAP0 or CAP1 structures. Human IFIT1 (hIFIT1), EIF4E and ILF3 in precipitates were detected by western blotting. Input shows 1/10^th^ (mIFIT1, EIF4E) and 1/30^th^ (hIFIT1) of the amount incubated with beads. (**c**) Affinity purification of luciferase-tagged human (hIFIT1) and murine (mIfit1) IFIT1 expressed in 293T cells on beads bearing 5′ OH, CAP, CAP0, or CAP1 RNA. (**d**) Binding of recombinant IFIT1 to capped RNAs. As in (**c**), but RNA-coated beads were incubated with recombinant His-tagged mouse Ifit1 (His-mIfit1), human His-hIFIT1 or human His-EIF4E and bound protein was quantified by western blotting. (**e**) Binding of recombinant His-tagged hIFIT1 and EIF4E to chemically synthesized, biotinylated RNA oligomers. Synthetic triphosphorylated RNAs with (CAP1) or without (CAP0) 2′O-methyl group on the first ribose were capped in vitro using recombinant vaccinia virus capping enzyme (see Materials and Methods). As control we used a synthetic RNA harbouring a 5′ hydroxyl group (OH). Synthetic RNAs were coupled to beads, incubated with recombinant proteins and bound proteins detected by western blotting. Input shows 1/10^th^ of the amount incubated with beads.

To identify proteins that bind capped RNA in a methylation-dependent manner we used unmethylated CAP-RNA and fully methylated CAP1-RNA as baits with IFN-treated HeLa cell lysates and quantified the captured proteins by LC-MS/MS. As expected [6], [7], most cellular proteins were significantly enriched in the CAP1-RNA bound fraction (Fig. 3a, Fig. S2c). The most notable exceptions were IFITs and the cellular 2′O-methyltransferase FTSJD2, both of which clearly favoured CAP-RNA (Fig. 3a, Fig. S2c and Fig. 1 c, d and f, g). We confirmed the MS data by a series of RNA precipitations followed by western blotting for endogenous proteins. Proteins associating to RNA in a 5′ independent manner, such as ILF3, precipitated similarly well regardless of the RNA used (Fig. 3b). Cap N7 methylation increased the association of EIF4E to RNA and methylation of the 2′O position did not impair precipitation efficiency. In accordance with the MS results, IFIT1 bound well to unmethylated CAP-RNA and CAP0-RNA (N7 methylated cap) but revealed reduced binding to CAP1-RNA (N7 methylated cap and 2′O methylated first ribos).

We next tested the contributions of individual cap methylation sites to IFIT1 binding. To this end, we measured binding of luciferase-tagged human and murine IFIT1 with either CAP-, CAP0- or CAP1-RNA. The unmethylated CAP-RNA bait captured more human or murine IFIT1 than either of the methylated RNAs (Fig. 3c). Furthermore, the analysis suggested that N7 methylation on the cap and 2′O methylation of the first ribose both contributed to the reduced binding of IFIT1 to RNA. Similarly, the precipitation efficiency of recombinant human and murine IFIT1 was reduced when capped *in vitro* transcribed RNAs were enzymatically methylated at the N7 and 2′O position (Fig. 3d) or when chemically synthesised RNAs with the same modifications were used (Fig. 3e). This was in contrast to EIF4E that showed prominent binding when CAP0- or CAP1-RNA was used (Fig. 3d, e). Collectively, these data suggest that human and murine IFIT1 have the capability to directly sense the methylation state of capped RNA.

### Antiviral activity of IFIT1 against 2′O methyltransferase-deficient viruses

**Figure 4:**
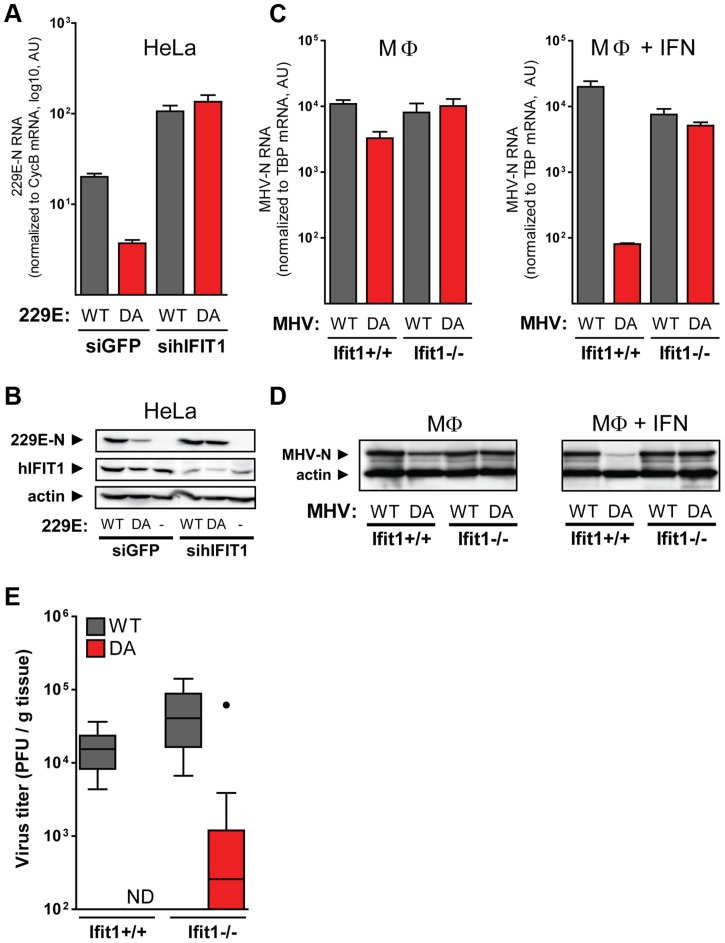
IFIT1 inhibits viral RNA and protein synthesis in cells infected with 2′O methyltransferase-deficient coronavirus. (**a–b**) HeLa cells were cotransfected for 48 h with an expression construct for the HCoV-229E receptor, human aminopeptidase N, and siRNAs targeting IFIT1 or the green fluorescent protein (GFP). Cells were then treated with 20 U IFN-α and infected with wild-type HCoV-229E (229E-WT; grey bars) or the 2′O methyltransferase-deficient HCoV-229E (D129A) mutant (229E-DA; red bars). Total RNA and protein were harvested 24 h post infection and analysed by quantitative RT-PCR (**a**) and western blotting (**b**), respectively. Quantitative RT-PCR data are from one of three representative experiments showing means ±SD for HCoV-229E nucleoprotein (229E-N) RNA after normalization to cyclin B (CycB) mRNA. (**c–d**) Bone marrow-derived macrophages (Mφ) derived from C57BL/6 (Ifit1^+/+^) and Ifit1-deficient (Ifit1^−/−^) mice were treated or not with 50 U of IFN-α for 2 h and infected with wild-type MHV (WT; grey bars) or 2′O methyltransferase-deficient MHV (DA; red bars). RNA and protein were harvested 8 h post infection and analysed by quantitative RT-PCR (**c**) and western blotting (**d**). Quantitative RT-PCR results are from one of three representative experiments, showing means ±SD for MHV nucleoprotein (MHV-N) RNA after normalization to the TATA-binding protein (TBP) mRNA. (**e**) Ifit1^+/+^ and Ifit1^−/−^ mice were infected intraperitoneally with 5,000 plaque-forming units of MHV WT (grey bars) or DA (red bars). Viral titers in the spleens of 12 mice per condition were measured 48 h after infection. Data are shown as Tukey box-whisker plots (ND, not detectable; outlier indicated as black dot).

Having established that IFIT1 binds directly to capped RNA and that methylation on the 2′O position of the first ribose markedly reduces binding, we tested the impact of IFIT1 on virus replication. Probably as a result of evolutionary pressure, most viruses that infect higher eukaryotes have evolved mechanisms to generate RNA that is methylated on both the N7 position of the guanosine cap and the 2′O position of the first ribose [9]. We therefore used wild-type human coronavirus (HCoV) 229E (229E-WT), which generates CAP1-RNA, and a mutant variant that has a single amino acid substitution (D129A) in the viral 2′O methyltransferase that is part of non-structural protein 16 (229E-DA), and consequently only produces CAP0-RNA [11]. IFN-α-treated HeLa cells infected with the 229E-DA mutant expressed significantly reduced levels of viral RNA and protein relative to those exposed to 229E-WT (Fig. 4a, b). Moreover, this effect was strictly dependent on IFIT1, since the two viruses replicated equally well in HeLa cells treated with siRNA against IFIT1 (Fig. 4a, b). Similar effects were observed in an analogous mouse model. Thus, when IFN-α treated macrophages (MΦs) from C57BL/6 (Ifit ^+/+^) mice were infected with a wild-type murine coronavirus (mouse hepatitis virus strain A59; MHV-WT) and a mutant strain carrying the equivalent amino acid substitution (D130A) in its 2′O methyltransferase [11], [17] (MHV-DA), the latter produced 100-fold less viral RNA and comparably reduced levels of viral protein (Fig. 4c, d). In contrast, when Ifit1-deficient MΦs were infected, no significant virus-dependent differences were observed, again pointing to a critical role for Ifit1 in restricting replication of MHV-DA. Note that the presence of Ifit1 itself did not increase IFN-α/β production (Fig. S6), suggesting a direct antiviral effect of Ifit1. We next assessed the impact of Ifit1 on virus growth *in vivo*. MHV-WT grew to high titres in the spleens of infected Ifit1^+/+^ mice, whereas no viral replication could be detected upon infection with MHV-DA (Fig. 4e). In agreement with the *in vitro* data, growth of MHV-DA was partially restored in Ifit1-deficient animals. These data suggest that IFIT1 has a central role in restraining the growth of 2′O methyltransferase-deficient coronaviruses *in vitro* and *in vivo*, which is compatible with the greater affinity of IFIT1 for non-2′O-methylated RNA cap structures. The data further imply that this role is conserved in mouse and human.

### IFIT1 specifically regulates the translation of 2′O unmethylated capped viral RNA

**Figure 5:**
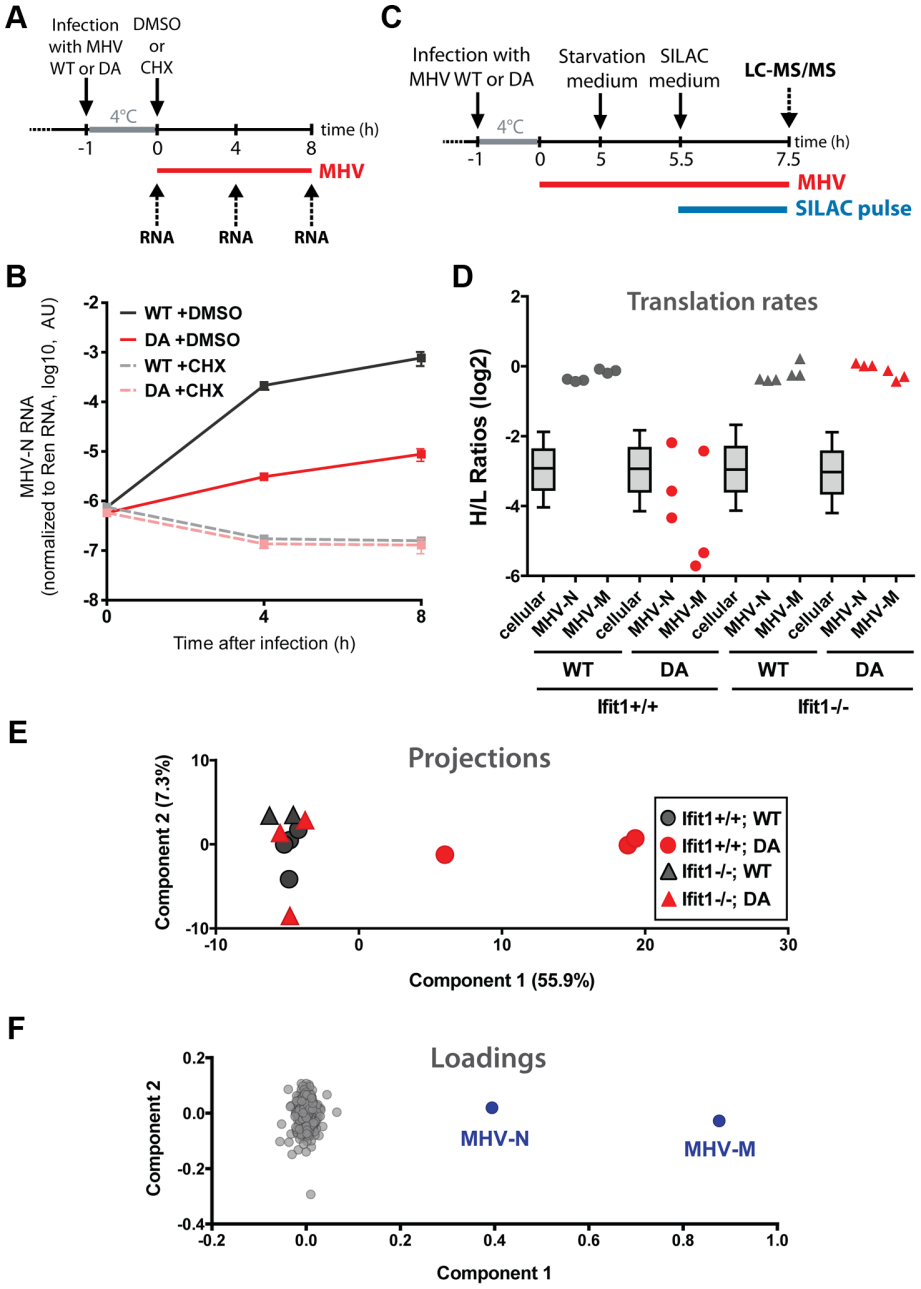
IFIT1 specifically blocks translation of 2′-O-unmethylated capped viral RNA. (**a**) Experimental design used to assess the stability of MHV RNA in infected cells. Bone marrow-derived macrophages (Mφ) from C57/BL6 mice were treated with 50 U of IFN-α for 2 h prior to infection with wild-type MHV (WT) or 2′O methyltransferase-deficient MHV (DA) at 4°C for 1 h. Directly after infection, cells were treated with 100 µg/ml cycloheximide (CHX) or DMSO. Total RNA was harvested at 0, 4, and 8 h post infection and analysed by quantitative RT-PCR. (**b**) MHV nucleoprotein (MHV-N) RNA in cells infected with MHV WT (grey) or DA mutant (red), treated with DMSO (solid lines) or CHX (dashed lines). Data from one representative experiment of three are depicted, showing means ±SD after normalization to a known amount of in vitro transcribed *Renilla* luciferase RNA (Ren) added to cell lysates. (**c**) Experimental design for pulsed SILAC coupled to mass spectrometry to determine relative changes in protein translation during infection. Macrophages from C75/BL6 (Ifit1^+/+^) and Ifit1-deficient (Ifit1^−/−^) mice grown in normal growth medium containing light (L) amino acids were infected at 4°C for 1 h with wild-type MHV (WT) or 2′O methyltransferase-deficient MHV (DA). Five hours post infection cells were incubated with starvation medium (lacking Lys and Arg) for 30 min, then SILAC medium containing heavy (H) labelled amino acids (Lys8, Arg10) was added, and 2 h later total protein lysate was prepared and subjected to LC-MS/MS analysis. (**d**) Translation rates for 721 cellular proteins, as determined by heavy (H) to light (L) ratios from LC-MS/MS, were plotted as box-whisker plots (whiskers from 10th to 90th percentile). Individual ratios for the MHV nucleoprotein (MHV-N) and membrane protein (MHV-M) in WT- (grey) and DA-infected (red) Ifit1^+/+^ (circles) and Ifit1^−/−^ (triangles) macrophages are plotted separately. Data are from three independent experiments. (**e,f**) Principal Component Analysis based on valid H/L ratios of all measurements from (**d**) showing clustering of the individual samples of the entire dataset (**e**). Panel (**f**) shows all proteins plotted for their contribution to the variation in components 1 and 2. MHV proteins are indicated in blue.

RNA capping is essential for a variety of cellular functions. The presence of a 5′ cap regulates mRNA export from the nucleus, protects RNAs from degradation and is necessary for efficient translation [7], [22]. An involvement of IFIT1 in nuclear-cytoplasmic transport is unlikely, given the exclusively cytoplasmic localisation of IFIT proteins and their negative effect on coronaviruses, which replicate in the cytoplasm. We therefore measured the stability of the RNAs generated by MHV-WT or MHV-DA in MΦs that had been stimulated with IFN-α. Since MHV-WT replicates significantly better than the mutant virus, we blocked virus replication by adding cycloheximide (CHX) shortly after infecting MΦs with the two viruses (Fig. 5a). CHX inhibits de novo synthesis of the viral polymerase, a prerequisite for transcription of viral RNA and thereby allows to normalise for viral transcripts in coronavirus infected cells. The abundance of viral transcripts 4 h and 8 h after infection was indistinguishable in CHX treated cells infected with MHV-WT and MHV-DA (Fig. 5b), suggesting that 2′O methylation of the first ribose does not affect the stability of the viral RNA within the timeframe of this experiment.

Many cellular antiviral defence mechanisms generally block translation of mRNA, thereby also severely inhibiting virus growth. To assess the global impact of Ifit1 on the translation machinery, we used pulsed stable isotope labelling in cell culture (SILAC) [23]. In pulsed SILAC, unlabelled cells are transferred to SILAC growth medium containing ^13^C- and ^15^N-labelled arginine (Arg10) and lysine (Lys8). Newly synthesized proteins incorporate the heavy label and pre-existing proteins remain in the light form, which allows to measure relative changes in the translation of individual proteins, regardless of the absolute amount of RNA present. We pulsed Ifit1^+/+^ and Ifit1^−/−^ MΦs infected for 5½ h with either MHV-WT or MHV-DA for 2 h with SILAC medium (Fig. 5c) and analysed infected cells by whole-proteome shotgun LC-MS/MS. We could reliably quantify 721 proteins in terms of heavy/light ratios in all three biological replicates tested. Heavy/light ratios of cellular proteins were comparable in Ifit1^+/+^ and Ifit1^−/−^ MΦs, irrespective of the virus used for infection (Fig. 5d, boxes), suggesting that neither the presence of Ifit1 nor infection with MHV-DA affected the overall rate of translation in the cells. The expression profiles of individual proteins known to be important in innate immune responses against viruses, such as the pattern recognition receptor RIG-I (DDX58), signalling molecules (STAT1, -2, -3), interferon-induced proteins (Ifi205b, Ifi35, Gvin1) and components of the major histocompatibility complex (H2-K1, H2-D1, Cd74), were similar in both cell types infected with either virus (Fig. S7). However, translation of viral nucleocapsid and membrane proteins was selectively reduced in Ifit1^+/+^ MΦs infected with MHV-DA (Fig. 5d and Fig. S7). Variation in large datasets can be best evaluated by principal-component analysis, which computes the variable with the greatest effect in a given dataset. This analysis revealed that Ifit1^+/+^ MΦs infected with MHV-DA showed the highest variation (Component 1 accounting for 55.9% of variation) as compared to all other conditions tested (Fig. 5e), and among all identified proteins, MHV proteins were mainly responsible for this variation (Fig. 5f). Taken together, these data indicate that synthesis of proteins encoded by viral RNAs lacking 2′O methylation on the first ribose is specifically inhibited by IFIT1. Expression of proteins encoded by fully methylated RNA, such as cellular mRNA or 2′O methylated viral RNA, is not affected by the activity of IFIT1.

### IFIT1 and translation factors compete for mRNA templates

**Figure 6:**
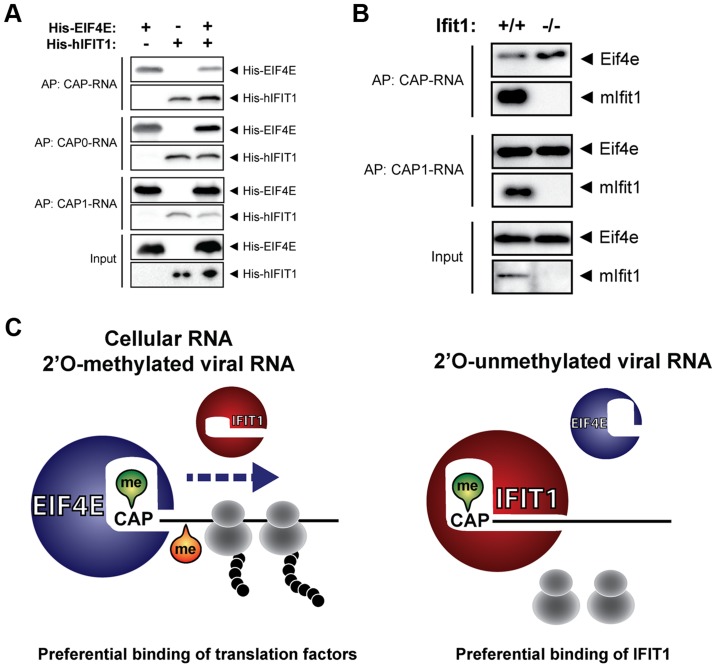
Competition between IFIT1 and translation factor EIF4E for mRNA templates. (**a**) Recovery of recombinant human EIF4E based on RNA affinity binding in the presence or absence of IFIT1. Streptavidin beads were coupled to 250 ng of the indicated RNA and mixed with 5 µg of recombinant His-tagged hIFIT1 and/or His-tagged EIF4E, as indicated. Bound proteins were analysed by western blotting with antibodies directed against the His-tag. (**b**) As in (**a**), except that RNA-coated beads were incubated with lysates of interferon-treated Ifit1^+/+^ and Ifit1^−/−^ mouse embryo fibroblasts. Bound proteins were analysed by western blotting with antibodies directed against murine Eif4e and mIfit1. (**c**) Proposed model for IFIT1-mediated translational inhibition of 2′O-unmethylated viral RNA. Capped and 2′O-methylated cellular and viral RNA is bound by EIF4E to initiate translation. Viral mRNA lacking 2′O methylation at the first ribose is recognized by IFIT1 which prevents binding of cellular factors required for efficient translation. The model is based on data presented here and elsewhere [16], [17], [19], [20].

Translation of cellular capped mRNA requires binding of the cap-binding protein EIF4E, which has a high affinity for methylated cap structures [7], [22]. Therefore, we tested whether IFIT1 could compete with EIF4E for binding to RNA template. We coupled limiting amounts of unmethylated CAP-RNA, N7-methylated CAP0-RNA and fully methylated CAP1-RNA to beads and tested whether the binding ability of recombinant EIF4E is altered by the presence of recombinant IFIT1. When we used CAP-RNA or CAP0-RNA, EIF4E binding to the beads was reduced by addition of IFIT1, suggesting that the two proteins compete for the RNA target (Fig. 6a). In contrast, when methylated CAP1-RNA was used the amount of EIF4E recovered was not affected by the presence of IFIT1. Competition between Eif4e and Ifit1 for capped RNA was also seen when total lysates of IFN-α-stimulated MEFs were used as inputs for experiments. Unmethylated CAP-RNA captured considerably more Eif4e from lysates of IFN-α treated Ifit1^−/−^ MEFs than from lysates of Ifit1^+/+^ MEFs (Fig. 6b). This difference disappeared when methylated CAP1-RNA was used as bait (Fig. 6b). We therefore conclude that IFIT1 competes with cellular translation initiation factors for mRNA, thereby selectively regulating translation based on the 5′ methylation status of the RNA templates present (Fig. 6c).

## Discussion

We previously identified IFIT1 as a nucleic acid-binding protein that recognises the 5′triphosphosphate present on genomes and transcripts of most negative-strand RNA viruses [16]. Here we show that, in addition, IFIT1 binds mRNAs that lack 2′O methylation on the first ribose, such as those produced by RNA viruses that replicate in the cytoplasm and are deficient in RNA cap-specific ribose-2′-O methyltransferase activity. This suggests that IFIT1 has a unique ability to recognize 5′ RNA modifications that are present on viral nucleic acids. Co-purification experiments with human IFIT proteins clearly show formation of a multiprotein complex comprising IFIT1, -2 and -3. Overexpression of single IFIT proteins, including IFIT1, only marginally affects viral growth [16], [17], suggesting that the cooperative action of IFIT proteins is required for full antiviral action. This is supported by loss-of-function experiments in cell culture and *in vivo* that show a requirement for Ifit2, which by itself does not bind CAP-RNA, to restrict viruses lacking 2′O methyltransferase activity [17], [18]. IFIT2 is known to bind to components of the cytoskeleton [24], which could allow intracellular trafficking of the IFIT complex to its sites of action. While some IFITs possess conserved biological activities in different species, e.g. human and murine IFIT1 which bind to PPP-RNA and unmethylated CAP-RNA, others appear to have evolved in a species-specific manner. We showed here that the yet uncharacterised murine interferon-induced Ifit1c binds to RNA-coated beads in an Ifit1-dependent manner, and we therefore propose that a corresponding Ifit complex with a different protein composition exists in mice.

Residues previously identified to be important for binding of the triphosphate moiety are also required for binding of unmethylated CAP-RNA by IFIT1, suggesting a conserved mechanism of RNA binding. In this context it is of interest to note that crystallographic analysis indicates that PPP-RNA binding to IFIT5, which shows high similarity to IFIT1, occurs in a fashion that is reminiscent of CAP-RNA binding by cap-binding proteins, in that the first two nucleotides are stacked by an aromatic phenylalanine [20]. However, the higher affinity of IFIT1 for unmethylated relative to fully methylated capped RNA is unusual among cellular proteins since 5′ methylation has so far been reported to increase the affinity of cellular proteins for RNA [7], a notion clearly supported by our RNA AP-MS data. Like its specific antiviral activity, this property of IFIT1 may only become apparent during infections with viruses that produce non-methylated RNA 5′ ends [25], [26]. We propose that IFIT1 acts as a molecular switch that allows selective translation based on the 5′ methylation state of the mRNA. The phenomenon of translational control by IFIT1 based on its differential affinity for the capped RNA is reminiscent of the 4E homologous protein (4EHP) in Drosophila and mice, which has been found to control translation by competing with EIF4E for the RNA cap structure, thereby regulating development-specific gene expression [27], [28]. Similarly, in our hands, IFIT1 does not associate directly with the translation machinery ([16] and data not shown), which again suggests that it perturbs translation through sequestration of viral RNA. Such a model is consistent with the high expression levels of IFIT proteins resulting from infections with viruses or treatment with IFN-α/β.

Rather than mediating general inhibition of translation, IFIT1 shows high selectivity for mRNAs that lack 5′ methylation. This is supported by pulsed SILAC experiments showing specific, IFIT1-dependent inhibition of translation of capped RNAs lacking 2′O methylation at the first ribose, such as those generated by MHV and HCoV mutants expressing inactive 2′O methyltransferase. Lower eukaryotes and viruses that infect them lack 2′O methylated CAP RNA [29]–[31], and the latter should be susceptible to the antiviral activity of IFITs. Consequently, the IFIT defence system is likely to contribute to a species barrier that puts selective pressure on viruses to generate 5′ methylated RNA. Our data provide a mechanistic rationale for why most viruses make considerable effort and dedicate part of their coding capacity to produce genomic and subgenomic RNAs with 5′-terminal ends that perfectly mimic those of cellular mRNAs, including fully methylated 5′-cap structures [9], [31]–[33]. Other viruses have evolved specific mechanisms to hide their uncapped/unmethylated 5′ ends, for example, by covalent binding of viral proteins to the 5′ end of viral RNAs and use of alternative strategies for translation initiation, thereby escaping IFIT1-based surveillance, which is centred on RNA 5′ end structures. Despite these viral strategies to generate host-like mRNAs, IFIT1 remains active against viruses that generate 5′ triphosphate RNA, most likely through translation-independent mechanisms. The ability of IFIT1 to target viral RNAs selectively allows the cell to specifically fight virus infections while pursuing an antiviral program aimed at destroying the intruding pathogen.

## Materials and Methods

### Ethics statement

All animal experiments were performed in accordance with Swiss federal legislation on animal protection and with the approval of the Animal Studies Committee of the Cantonal Veterinary Office (St. Gallen, Switzerland), license nr. SG 11/03.

### Reagents, cells and viruses

Interferon-α (IFN-α A/D) was a kind gift from Peter Stäheli. Expression constructs for human and murine IFIT proteins [16], [20] and the human aminopeptidase N (APN) were described previously. Products tagged with *Renilla* luciferase were expressed from constructs obtained by Gateway cloning into pCDNA-REN-NT-GW (a kind gift from Albrecht v. Brunn). For expression in bacteria, human EIF4E cDNA was cloned into pETG10A-GW [16]. Recombinant IFIT proteins and human EIF4E were expressed in *E. coli* and purified using HisPur Ni-NTA resin (Thermo Scientific). Streptavidin-agarose beads were obtained from Novagen. Polyclonal antibodies directed against human and mouse IFIT1 were described previously [16]. The antibody against MHV nucleoprotein (MHV-N556) was kindly donated by Stuart Siddell. Primary antibodies against ILF-3 (Sigma; HPA001897), the nucleoprotein of HCoV-229E (Ingenasa; mAb 1H11) and EIF4E (Cell Signaling; C46H6) were obtained from commercial sources. For western blot analysis we used horseradish peroxidase (HRP)-coupled antibodies specific for actin (Santa Cruz; sc-47778), the His-tag (Santa Cruz; sc-8036) or the c-Myc-tag (Roche; 1667149), and HRP-coupled secondary antibodies (Jackson ImmunoResearch). All cell lines used (293T, HeLa, Vero-E6, Huh7, L929, 17Clone1, and Ifit1^+/+^ and Ifit1^−/−^ mouse embryonic fibroblasts) were described previously [11], [16], and were maintained in DMEM (PAA Laboratories) containing 10% fetal calf serum (PAA Laboratories) and antibiotics (100 U/ml penicillin, 100 µg/ml streptomycin). DMEM medium containing antibiotics, 10 mM L-glutamine, 10% dialyzed fetal calf serum (PAA Laboratories) and 84 mg/L ^13^C_6_^15^N_4_ L-arginine and 146 mg/L ^13^C_6_^15^N_2_ lysine (Cambridge Isotope Laboratories) was used for SILAC experiments. Murine bone marrow-derived macrophages were generated *in vitro* by cultivating bone marrow from mouse femur and tibia in DMEM supplemented with 10% (v/v) fetal calf serum, 5% (v/v) horse serum, 10 mM HEPES pH 7.4, 1 mM sodium pyruvate, 10 mM L-glutamine and 20% (v/v) L929 cell-conditioned medium (containing macrophage colony-stimulating factor) for 6 days. Reagents for transfection with plasmid DNA (Nanofectin) or siRNA duplexes (siRNA Prime) were obtained from PAA Laboratories. Wild-type and 2′-O-methyltransferase-deficient recombinant coronaviruses [mouse hepatitis virus strain A59 (MHV) and human coronavirus 229E (HCoV-229E) [11]], Sendai virus, RVFV Clone13 [34] and VSV-M2 (mutant VSV with the M51R substitution in the matrix protein) [35] have been described previously. Duplex siRNAs targeting human IFIT1 [sense#1: r(CAUGGGAGUUAUCCAUUGA)dTdT; antisense#1: r(UCAAUGGAUAACUCCCAUG)dTdA; sense#2: r(CCUUGGGUUCGUCUACAAA)dTdT, antisense#2: r(UUUGUAGACGAACCCAAGG)dAdG] and the green fluorescent protein [sense: 5′ r(AAGCAGCACGACUUCUUCAAGU)dT 3′; antisense 5′ r(CUUGAAGAAGUCGUGCUGCUUU)dT 3′] were synthesized by the Core Facility at the MPI of Biochemistry.

### Capping and methylation of in vitro transcribed RNA

Triphosphorylated PPP-RNA was synthesized by in vitro transcription with SP6 or T7 polymerase (RiboMAX Large Scale RNA Production Systems; Promega), in the presence or absence of biotin-16-UTP (Enzo), from plasmids encoding antisense 7SK RNA (7SK-as) [13] or *Renilla* luciferase (pRL-SV40; Promega), and purified by ammonium-acetate isopropanol precipitation. Aliquots of PPP-RNA were then mock-treated, dephosphorylated with alkaline phosphatase (FastAP; Fermentas), or modified with different 5′ cap structures using the ScriptCap 2′-O-Methyltransferase and m7G Capping System (CellScript) according to the manufacturer's instructions. Briefly, 20-µg samples of RNA were heat-denatured at 65°C for 5 min, cooled on ice, then incubated with ScriptCap Buffer in the presence of 500 µM GTP, 100 µM SAM, 100 U 2′-O-methyltransferase (VP39), 10 U Vaccinia Capping Enzyme (VCE) and 40 U RNase inhibitor for 1 h at 37°C. Capped RNAs were further treated with FastAP to dephosphorylate any residual PPP-RNA, and then column-purified using the NucleoSpin RNA II kit (Macherey-Nagel). To add radioactively labelled methyl groups to in vitro transcribed RNA, 500 ng of each RNA was incubated with 100 U 2′-O-methyltransferase or 10 U of VCE in 0.5 µM S-adenosylmethionine and 1.4 µM S-[^3^H-methyl]-adenosylmethionine (78 Ci/mmol; Perkin-Elmer) for 1 h at 37°C. Reactions were purified on SigmaSpin Post-Reaction Clean-Up columns (Sigma) and eluates were mixed with 2 ml Ultima Gold scintillation fluid for measurement of ^3^H incorporation with a Packard Tri-Carb liquid scintillation counter (Perkin Elmer).

### Generation and capping of chemically synthesized RNA oligomers

Capped m7Gppp-oligoribonucleotides matching the first 22 nucleotides of the 5′ untranslated region of Severe Acute Respiratory Syndrome Coronavirus HKU-39849 were prepared by adding N7-methylated cap structures to chemically synthesized RNA oligomers with a 3′-terminal C6 amino linker. A triphosphorylated RNA oligomer [PPP-r(AUAUUAGGUUUUUACCUACCC)-NH_2_) and a corresponding 2′O-ribose methylated RNA-oligomer [PPP-r(AmUAUUAGGUUUUUACCUACCC)-NH_2_] were ordered from ChemGenes Corporation (Wilmington, MA, USA) and capped as described above using the m7G Capping System (CellScript). Capped RNA oligomers were then HPLC-purified, biotinylated with biotin-N-hydroxysuccinimide ester (Epicentre) according to the manufacturer's instructions and again HPLC-purified. As control we used a corresponding 3′-terminal biotinylated and HPLC-purified oligoribonucleotide harbouring a 5′ hydroxyl group [OH-r(AUAUUAGGUUUUUACCUACCCU)-biotin].

### Identification and quantitation of RNA-binding proteins

For quantitative purification of RNA-binding proteins, streptavidin affinity resin was first incubated with 1-µg aliquots of biotin-labelled OH-RNA, PPP-RNA, CAP-RNA, CAP0-RNA or CAP1-RNA (all 7SK-antisense) in TAP buffer [50 mM Tris pH 7.5, 100 mM NaCl, 5% (v/v) glycerol, 0.2% (v/v) Nonidet-P40, 1.5 mM MgCl_2_ and protease inhibitor cocktail (EDTA-free, cOmplete; Roche)] in the presence of 40 U RNase inhibitor (Fermentas) for 60 min at 4°C on a rotary wheel. Control or RNA-coated beads were then incubated with 2-mg samples of HeLa cell lysate for 60 min, washed three times with TAP buffer, and twice with TAP buffer lacking Nonidet-P40 to remove residual detergent. Three independent affinity purifications were performed for each RNA. Bound proteins were dentatured by incubation in 6 M urea-2 M thiourea with 1 mM DTT (Sigma) for 30 min and alkylated with 5.5 mM iodoacetamide (Sigma) for 20 min. After digestion with 1 µg LysC (WAKO Chemicals USA) at room temperature for 3 h, the suspension was diluted in 50 mM ammonium bicarbonate buffer (pH 8). The beads were removed by filtration through 96-well multiscreen filter plates (Millipore, MSBVN1210), and the protein solution was digested with trypsin (Promega) overnight at room temperature. Peptides were purified on stage tips with three C18 Empore filter discs (3M) and analyzed by mass spectrometry as described previously [36]. Briefly, peptides were eluted from stage tips and separated on a C18 reversed-phase column (Reprosil-Pur 120 C18-AQ, 3 µM, 150×0.075 mm; Dr. Maisch) by applying a 5% to 30% acetonitrile gradient in 0.5% acetic acid at a flow rate of 250 nl/min over a period of 95 min, using an EASY-nanoLC system (Proxeon Biosystems). The nanoLC system was directly coupled to the electrospray ion source of an LTQ-Orbitrap XL mass spectrometer (Thermo Fisher Scientific) operated in a data dependent mode with a full scan in the Orbitrap cell at a resolution of 60,000 with concomitant isolation and fragmentation of the ten most abundant ions in the linear ion trap.

### Affinity purification of luciferase-tagged and recombinant proteins

N-terminally *Renilla* luciferase-tagged proteins were transiently expressed in 293T cells. Three micrograms of each construct were transfected into 6×10^6^ cells using 9.6 µl nanofectin (PAA Laboratories) in 10-cm dishes according to the manufacturer's instructions. After 24 h, the medium was removed, and cells were lysed in ice-cold TAP lysis buffer. An aliquot (10%) of the lysate was removed to determine input luciferase activity. The rest was added to streptavidin-agarose beads coated with 250 ng of RNA as described above, and incubated on a rotary wheel at 4°C for 60 min. Beads were washed three times and resuspended in 50 µl TAP buffer. Luciferase activities present in the suspension and in the input lysate were assayed in Renilla reaction buffer (100 mM K_3_PO_4_, 500 mM NaCl, 1 mM EDTA, 25 mM thiourea) containing 10 µM coelenterazine as substrate. The reactions were performed in triplicate and results were quantified using an Infinite 200 PRO series microplate reader (Tecan). For affinity purification of recombinant proteins with different RNAs, 50 to 250 ng of biotinylated RNA were coupled to streptavidin-agarose beads for 60 min at 4°C. Beads were washed three times with TAP buffer and incubated with recombinant His-tagged proteins for 60 min at 4°C. After three washes beads were boiled in Laemmli buffer for 10 min at 95°C and subjected to SDS-PAGE and Western Blot analysis.

### Real-time RT-PCR

Total RNA was isolated using the NucleoSpin RNA II kit (Macherey-Nagel), including on-column DNase digestion, and 200 to 500 ng of RNA was reverse transcribed with the RevertAid H Minus First Strand cDNA Synthesis Kit (Fermentas). RNA levels were then quantified by real-time RT-PCR using the QuantiTect SYBR Green RT-PCR kit (Qiagen) and a CFX96 Touch Real-Time PCR Detection System (BioRad). Each cycle consisted of 15 sec at 95°C, 30 sec at 50°C and 30 sec at 72°C, followed by melting curve analysis. Primer sequences were as follows: *Renilla* luciferase (5′-CGAAAGTTTATGATCCAGAAC-3′ and 5′-AATCATAATAATTAATAAATG-3′), hCycB (5′-CAGCAAGTTCCATCGTGTCATCAAGG-3′ and 5′-GGAAGCGCTCACCATAGATGCTC-3′), mTBP (5′-CCTTCACCAATGACTCCTATGAC-3′ and 5′- CAAGTTTACAGCCAAGATTCA-3′), mIFN-β (5′-ATGGTGGTCCGAGCAGAGAT-3′ and 5′-CCACCACTCATTCTGAGGCA-3′), MHV-N (5′-GCCTCGCCAAAAGAGGACT-3′ and 5′- GGGCCTCTCTTTCCAAAACAC-3′), 229E-N (5′-CAGTCAAATGGGCTGATGCA-3′ and 5′- AAAGGGCTATAAAGAGAATAAGGTATTCT-3′), mIfit1 (5′- CCATAGCGGAGGTGAATATC-3′ and 5′- GGCAGGACAATGTGCAAGAA-3′), mIfit1c (5′-AATCAGAAGAGGCAGCCATC-3′ and 5′-CATGGCTTCACTTGTGTTCC-3′), mIfit2 (5′-TCAGCACCTGCTTCATCCAA-3′ and 5′-CACCTTCGGTATGGCAACTT-3′), and mIfit3 (5′-GCTGCGAGGTCTTCAGACTT-3′ and 5′-TGGTCATGTGCCGTTACAGG-3′).

### Virus infection experiments in cell culture and in vivo

C57BL/6 mice were obtained from Charles River Laboratories (Sulzfeld, Germany), and *Ifit1^−/−^* mice have been described [16], [17]. Mice were maintained in individually ventilated cages and used at 6 to 9 weeks of age. All animal experiments were performed in accordance with Swiss federal legislation on animal protection and with the approval of the Animal Studies Committee of the Cantonal Veterinary Office (St. Gallen, Switzerland). Wild-type and *Ifit1^−/−^* mice (kindly provided by Michael Diamond) were injected intraperitoneally with 5,000 plaque-forming units of MHV. Virus titers in samples of spleens removed and frozen 48 h post infection were assessed by standard plaque assay on L929 cells. Bone marrow-derived macrophages or mouse embryo fibroblasts (1 to 5×10^5^ cells) were treated or not with IFN-α and infected with the indicated viruses at a multiplicity of infection (MOI) of 5. For synchronised infection, cells were infected with virus on ice and pre-warmed DMEM growth medium was added 1 h later. To quantify the effects of siRNA-mediated knockdown of IFIT1, aliquots of 10^5^ HeLa cells that had been transfected for 48 h with 15 pmol siRNA and 500 ng expression plasmid for human APN using the siRNA Prime reagent (PAA Laboratories) according to the manufacturer's instructions, were pretreated with IFN-α as indicated and infected with HCoV-229E at an MOI of 1 for 24 h.

### Pulsed SILAC and mass spectrometry

For pulsed SILAC, mouse macrophages labelled with heavy isotopes (see above) were lysed in SDS lysis buffer (50 mM Tris pH 7.5, 4% sodium dodecyl sulfate). The lysate was then heated for 5 min at 95°C, sonicated for 15 min with a Bioruptor (Diagenode) and centrifuged for 5 min at 16,000× *g* at room temperature. Protein concentration was determined by Lowry assay (DC Protein Assay, BioRAD), and 50-µg aliquots were reduced with 10 mM DTT for 30 min, alkylated with 55 mM IAA for 20 min at room temperature, and precipitated with 80% acetone for 3 h at −20°C. After centrifugation for 15 min at 16,000× *g* at 4°C, pellets were washed with 80% acetone, dried for 30 min at room temperature and dissolved in 6 M urea-2 M thiourea. Proteins were digested with LysC and trypsin at room temperature and peptides were purified on stage tips and analysed by LC-MS/MS using a Easy nano LC system coupled to a Q Exactive mass spectrometer (Thermo Fisher Scientific). Peptide separation was achieved on a C18-reversed phase column (Reprosil-Pur 120 C18-AQ, 1.9 µM, 200×0.075 mm; Dr. Maisch) using a 95-min linear gradient of 2 to 30% acetonitrile in 0.1% formic acid. The mass spectrometer was set up to run a Top10 method, with a full scan followed by isolation, HCD fragmentation and detection of the ten most abundant ions per scan in the Orbitrap cell.

### Bioinformatic analysis

Raw mass-spectrometry data were processed with MaxQuant software versions 1.2.7.4 and version 1.3.0.5 [37] using the built-in Andromeda search engine to search against human and mouse proteomes (UniprotKB, release 2012_01) containing forward and reverse sequences, and the label-free quantitation algorithm as described previously [36], [38]. In MaxQuant, carbamidomethylation was set as fixed and methionine oxidation and N-acetylation as variable modifications, using an initial mass tolerance of 6 ppm for the precursor ion and 0.5 Da for the fragment ions. For SILAC samples, multiplicity was set to 2 and Arg10 and Lys8 were set as heavy label parameters. Search results were filtered with a false discovery rate (FDR) of 0.01 for peptide and protein identifications. Protein tables were filtered to eliminate the identifications from the reverse database and common contaminants.

In analyzing mass spectrometry data from RNA affinity purifications, only proteins identified on the basis of at least two peptides and a minimum of three quantitation events in at least one experimental group were considered. Label-free quantitation (LFQ) protein intensity values were log-transformed and missing values filled by imputation with random numbers drawn from a normal distribution, whose mean and standard deviation were chosen to best simulate low abundance values. Significant interactors of RNAs with different 5′ end structures were determined by multiple equal variance t-tests with permutation-based false discovery rate statistics [39]. We performed 250 permutations and the FDR threshold was set between 0.02 and 0.1. The parameter *S*_0_ was empirically set between 0.2 and 1, to separate background from specifically enriched interactors.

For data analysis from pulsed SILAC experiments, we used log-transformed heavy to light protein ratios. Only proteins with valid values were considered for analysis, and normalized by dividing by the row median. Profile plots were generated using LFQ intensities of log-transformed heavy-labelled protein intensities. We excluded proteins containing less than 10 valid values in all 12 measurements, and missing values were filled by imputation. LFQ intensities were then normalized by dividing by the row median.

Results were plotted using R (www.R-project.org) and GraphPad Prism version 5.02. Multiple sequence alignments were generated with ClustalW (http://www.ebi.ac.uk/Tools/msa/clustalw2/).

### Structural modelling

A homology model of human IFIT1 was obtained with MODELLER [40] using the X-ray structure of human IFIT5 (4HOQ) as a structural template [20]. A pairwise sequence alignment was generated with ClustalW (http://www.ebi.ac.uk/Tools/msa/clustalw2/) and further refined with MODELLERs align2d. Human IFIT1 and IFIT5 share approximately 75.6% sequence similarity, with 57.2% of all residues being identical. Cavity volumes in both structures were calculated in a two-step process with the rolling probe method using 3V [41]. First, a solvent-excluded volume was calculated for each structure using a probe radius of 1.5 Å (corresponding to water). A larger probe size of 5 Å was used to calculate so-called “shell volumes”. The solvent-accessible cavity volumes were obtained by subtraction of each solvent-excluded volume from the corresponding shell volume.

## Supporting Information

Figure S1 **Generation of 5′end modified in-vitro transcribed RNA.** (**a**) Schematic overview of synthesis of the biotinylated RNA used in this study. 5′ triphosphorylated (PPP-) 7SK-antisense RNA obtained by in vitro transcription with SP6 polymerase was modified enzymatically at the 5′ end by incubating with alkaline phosphatase (AP) to remove 5′ phosphates (OH-RNA), with recombinant Vaccinia virus capping enzyme (VCE) to produce unmethylated capped RNA (CAP-RNA), with VCE in the presence of S-adenosyl methionine (SAM) to generate N7-methylated capped RNA (CAP0-RNA), or with VCE and recombinant Vaccinia virus 2′O methyltransferase (VP39) in the presence of SAM to generate N7-methylated capped RNA methylated at the 2′O position of the first ribose (CAP1-RNA) [43],[44]. (**b**) Agarose gel image showing 200 ng of in vitro transcribed, biotinylated RNA following the enzymatic treatments depicted in (**a**). (**c**) Evaluation of the N7- and 2′O-methylation efficiency of recombinant Vaccinia virus enzymes. Capped RNAs produced as in (**a**) were incubated either with VCE or VP39 in the presence of ^3^H-labeled SAM, and the incorporation efficiency was measured by scintillation counting. ^3^H-labeled methyl groups were transferred from SAM only if the RNA had not previously been methylated (N7-methylation of CAP-RNA, and 2′O methylation of CAP0-RNA), showing that methylation of RNA by both VCE and VP39 was maximally efficient.
(TIF)

Figure S2 **RNA affinity purifications from HeLa cell lysates.** (**a**) Heatmap of all proteins identified in RNA affinity purifications from HeLa cell lysates. Hierarchical clustering of proteins was performed on logarithmic LFQ protein intensities using Euclidean distances. The colour code represents LFQ intensities in rainbow colours (see colour scale). (**b**) Heatmap showing hierarchical clustering (Euclidean distances) of interactors that were significantly enriched (see Materials and Methods) in fractions bound by at least one RNA with a modified 5′ end structure (compared to OH-RNA). The plot shows means of Z-score transformed logarithmic LFQ intensities. Blue colours indicate Z-score <0, red colours indicate Z-score >0, white indicates Z-score = 0. The saturation threshold is set at -2.25 and +2.25. Asterisks indicate the IFIT complex. (**c**) Volcano plots showing enrichment (ratio of LFQ protein intensities; x-axis) and p-values (t-test; y-axis) of CAP1-RNA to CAP-RNA. Data are from three independent affinity purifications. Significantly enriched interactors (see Materials and Methods) are separated from background proteins (blue dots) by a hyperbolic curve (dotted line). Among the significant interactors, IFIT proteins and FTSJD2 (red) are highlighted.
(TIF)

Figure S3 **RNA affinity purifications from lysates of mouse embryo fibroblasts.** (**a–b**) As in Fig. S2, but showing proteins identified in RNA affinity purifications from mouse embryo fibroblasts. In (**b**) the saturation threshold is set at −1. 5 and +1. 5. The asterisk indicates the Ifit complex.
(TIF)

Figure S4 **Characterisation of the murine IFIT complex.** (**a**) Expression of Ifit genes in wild-type (Ifit1^+/+^) and Ifit1-deficient (Ifit1^−/−^) mouse embryonic fibroblasts (MEFs). MEFs were left untreated, treated with 1000 U/ml IFN-α, or infected with Rift Valley fever virus Clone13 or a mutant version of vesicular stomatitis virus (VSV-M2) at a multiplicity of infection of 1 or 0.01, respectively. Sixteen hours later RNA was analysed by quantitative RT-PCR for mIfit1, mIfit1c, mIfit2 and mIfit3. In each case, one representative experiment of three is shown, with means ±SD after normalization to the TATA-binding protein (TBP) mRNA. (**b**) Heatmap of selected proteins identified in RNA affinity purifications from cell lysates of Ifit1^+/+^ and Ifit1^−/−^ MEFs. The plot shows the means of log-transformed label-free quantitation protein intensities in rainbow colours (see colour scale). (**c**) Alignment of murine and human IFIT proteins using ClustalW. (**d**) Matrix showing amino acid similarity (based on ClustalW alignment) of all murine and human IFIT proteins. Percent similarity is indicated as color coded from white to red, and the exact similarity is shown within each element of the matrix.
(TIF)

Figure S5 **Comparison of the RNA binding cavities of IFIT5 and IFIT1.** Sections of surface representations of the solvent-accessible surfaces of IFIT5 (top) and IFIT1 (bottom) are shown, with PPP-RNA bound as in IFIT5 (stick representation, superimposed on IFIT1), and the corresponding cavity volumes V calculated as described in Materials and Methods. In our calcuations, the main RNA-binding cavity in IFIT5 has volume of 11881 Å^3^. The calculated volume of the corresponding cavity of the modelled IFIT1, at 12627 Å^3^, is about 700 Å^3^ larger.
(TIF)

Figure S6 **Induction of interferon-β in wild-type and Ifit1-deficient mouse cells.** Interferon-stimulated bone marrow-derived macrophages (MΦs) from C57/BL6 (Ifit1^+/+^) or Ifit1-deficient (Ifit1^−/−^) mice were left untreated, or infected with wild-type MHV (WT), 2′O-methyltransferase-deficient MHV (DA), or Sendai virus (SeV). Twelve hours later total RNA was harvested and analysed by quantitative RT-PCR for interferon β (IFN-β) mRNA. Data from three independent experiments showing fold change relative to untreated cells (mean ±SD) after normalization to the TATA-binding protein (TBP) mRNA.
(TIF)

Figure S7 **Translation profiles of individual proteins in MHV-infected macrophages.** Translation profiles based on pulsed SILAC of macrophages from C75/BL6 (Ifit1^+/+^) and Ifit1-deficient (Ifit1^−/−^) mice infected with wild-type MHV (WT) or 2′O methyltransferase-deficient MHV (DA) as shown in Fig. 5. The profile plot shows normalized LFQ intensities of heavy proteins, representing a total number of 451 proteins labelled during the 2 h pulse period. Data show average LFQ intensities from three independent replicates. Selected profiles are coloured and represent MHV proteins and cellular proteins involved in immune responses.
(TIF)

Table S1 **Quantitative MS data from RNA affinity purifications with HeLa cell lysates.** Proteins identified by LC-MS/MS from lysates of HeLa cells upon affinity purification with different RNA baits. Table contains log-transformed and imputed label-free quantification (LFQ) intensities of all identified proteins. Significantly enriched proteins, p values and mean differences from t-test based analyses are indicated.
(XLSX)

Table S2 **Quantitative MS data from RNA affinity purifications with MEF lysates.** Proteins identified by LC-MS/MS from lysates of mouse embryo fibroblasts (MEF) upon affinity purification with different RNA baits. Table contains log-transformed and imputed label-free quantification (LFQ) intensities of all identified proteins. Significantly enriched proteins, p values and mean differences from t-test based analyses are indicated.
(XLSX)
