# Supplementary figures and images for: Sequestration by IFIT1 Impairs Translation of 2′O-unmethylated Capped RNA
Source: PLoS Pathog. 2013 Oct 3;9(10):e1003663. doi: 10.1371/journal.ppat.1003663 (PMC3789756; doi:10.1371/journal.ppat.1003663)

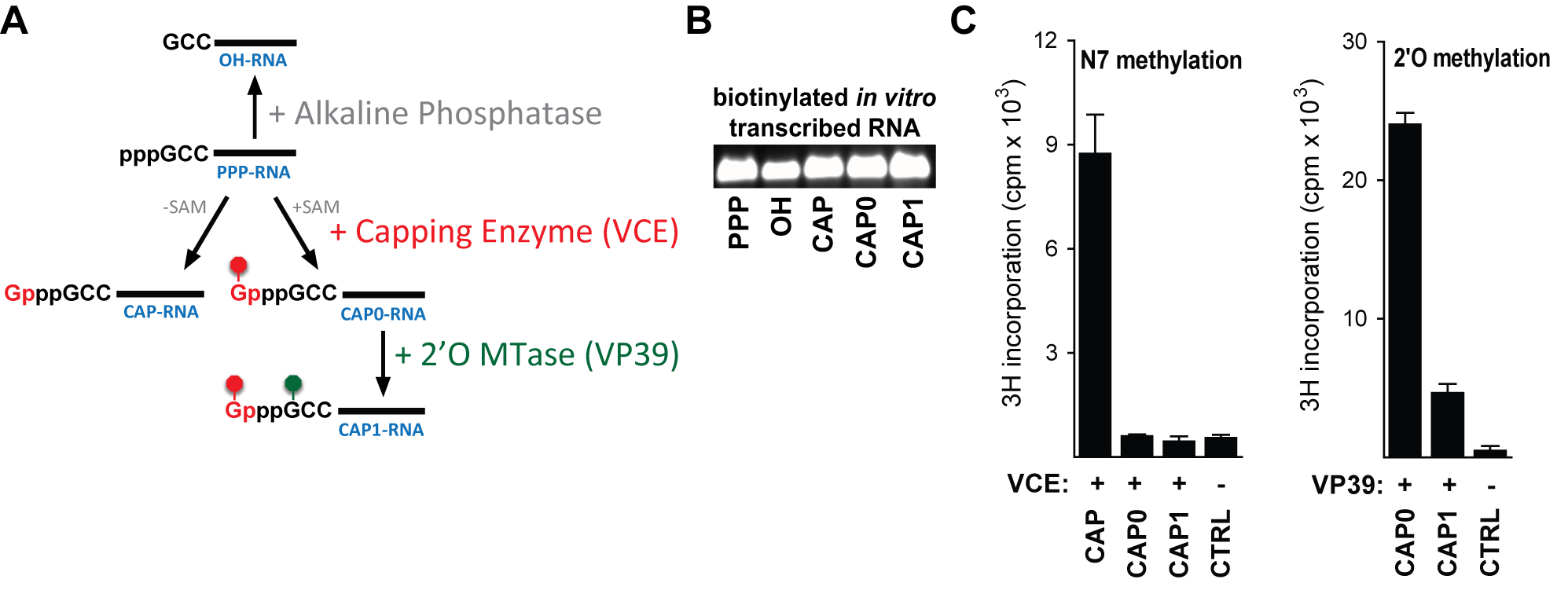

Supplement: Figure S1 — Generation of 5′end modified in-vitro transcribed RNA. (a) Schematic overview of synthesis of the biotinylated RNA used in this study. 5′ triphosphorylated (PPP-) 7SK-antisense RNA obtained by in vitro transcription with SP6 polymerase was modified enzymatically at the 5′ end by incubating with alkaline phosphatase (AP) to remove 5′ phosphates (OH-RNA), with recombinant Vaccinia virus capping enzyme (VCE) to produce unmethylated capped RNA (CAP-RNA), with VCE in the presence of S-adenosyl methionine (SAM) to generate N7-methylated capped RNA (CAP0-RNA), or with VCE and recombinant Vaccinia virus 2′O methyltransferase (VP39) in the presence of SAM to generate N7-methylated capped RNA methylated at the 2′O position of the first ribose (CAP1-RNA) [43],[44]. (b) Agarose gel image showing 200 ng of in vitro transcribed, biotinylated RNA following the enzymatic treatments depicted in (a). (c) Evaluation of the N7- and 2′O-methylation efficiency of recombinant Vaccinia virus enzymes. Capped RNAs produced as in (a) were incubated either with VCE or VP39 in the presence of 3H-labeled SAM, and the incorporation efficiency was measured by scintillation counting. 3H-labeled methyl groups were transferred from SAM only if the RNA had not previously been methylated (N7-methylation of CAP-RNA, and 2′O methylation of CAP0-RNA), showing that methylation of RNA by both VCE and VP39 was maximally efficient. (TIF) [file ppat.1003663.s001.tif]

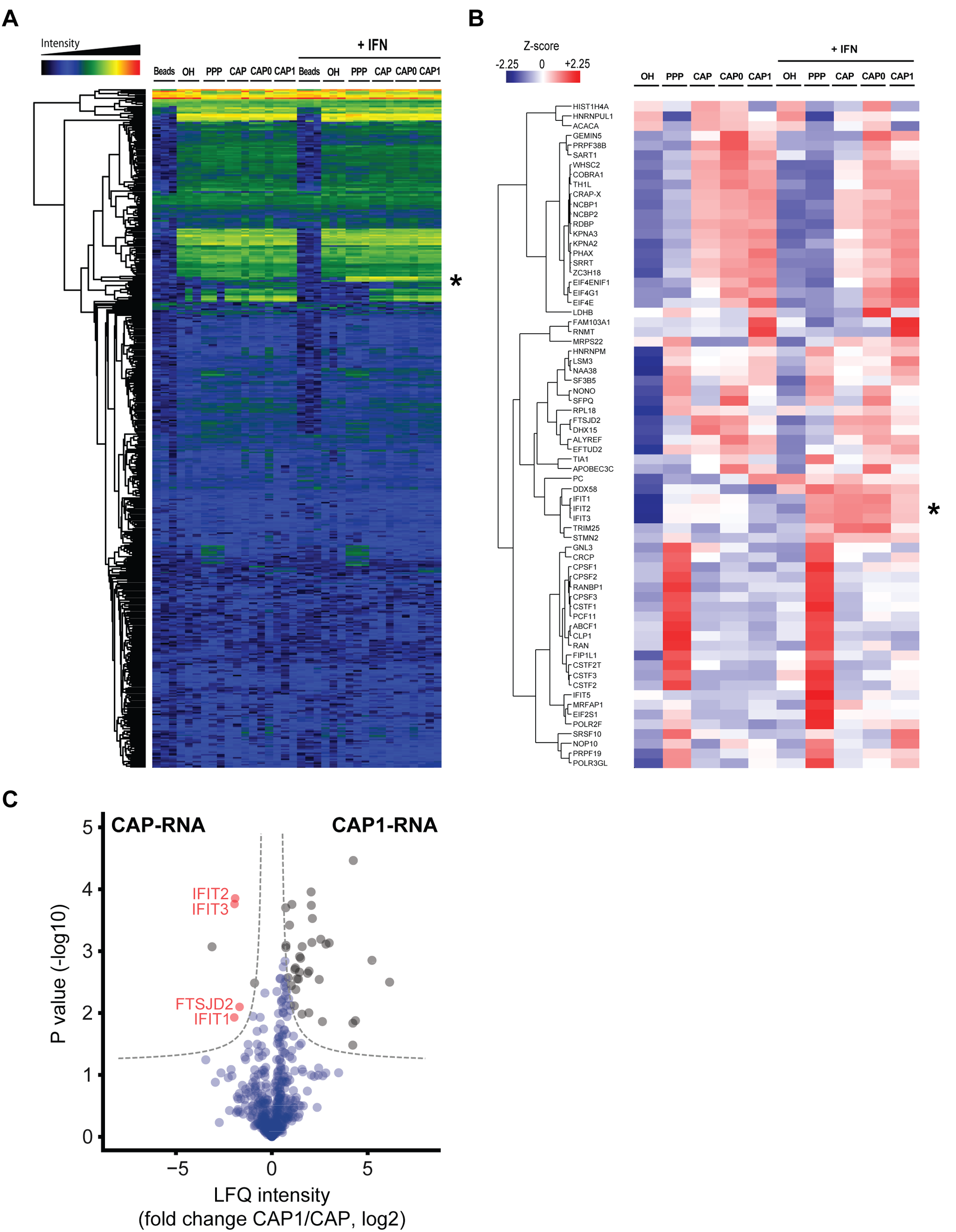

Supplement: Figure S2 — RNA affinity purifications from HeLa cell lysates. (a) Heatmap of all proteins identified in RNA affinity purifications from HeLa cell lysates. Hierarchical clustering of proteins was performed on logarithmic LFQ protein intensities using Euclidean distances. The colour code represents LFQ intensities in rainbow colours (see colour scale). (b) Heatmap showing hierarchical clustering (Euclidean distances) of interactors that were significantly enriched (see Materials and Methods) in fractions bound by at least one RNA with a modified 5′ end structure (compared to OH-RNA). The plot shows means of Z-score transformed logarithmic LFQ intensities. Blue colours indicate Z-score <0, red colours indicate Z-score >0, white indicates Z-score = 0. The saturation threshold is set at -2.25 and +2.25. Asterisks indicate the IFIT complex. (c) Volcano plots showing enrichment (ratio of LFQ protein intensities; x-axis) and p-values (t-test; y-axis) of CAP1-RNA to CAP-RNA. Data are from three independent affinity purifications. Significantly enriched interactors (see Materials and Methods) are separated from background proteins (blue dots) by a hyperbolic curve (dotted line). Among the significant interactors, IFIT proteins and FTSJD2 (red) are highlighted. (TIF) [file ppat.1003663.s002.tif]

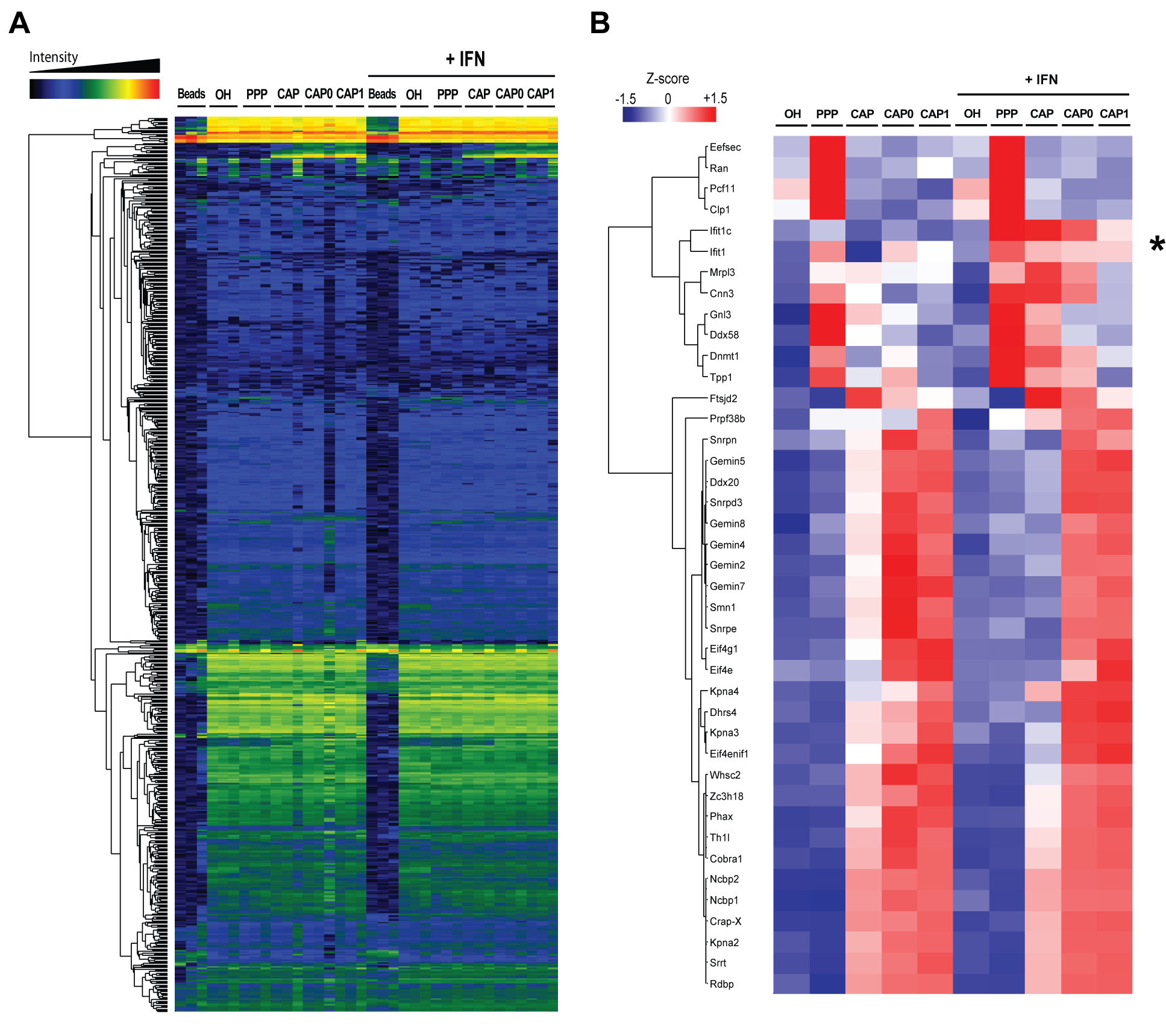

Supplement: Figure S3 — RNA affinity purifications from lysates of mouse embryo fibroblasts. (a–b) As in Fig. S2, but showing proteins identified in RNA affinity purifications from mouse embryo fibroblasts. In (b) the saturation threshold is set at −1. 5 and +1. 5. The asterisk indicates the Ifit complex. (TIF) [file ppat.1003663.s003.tif]

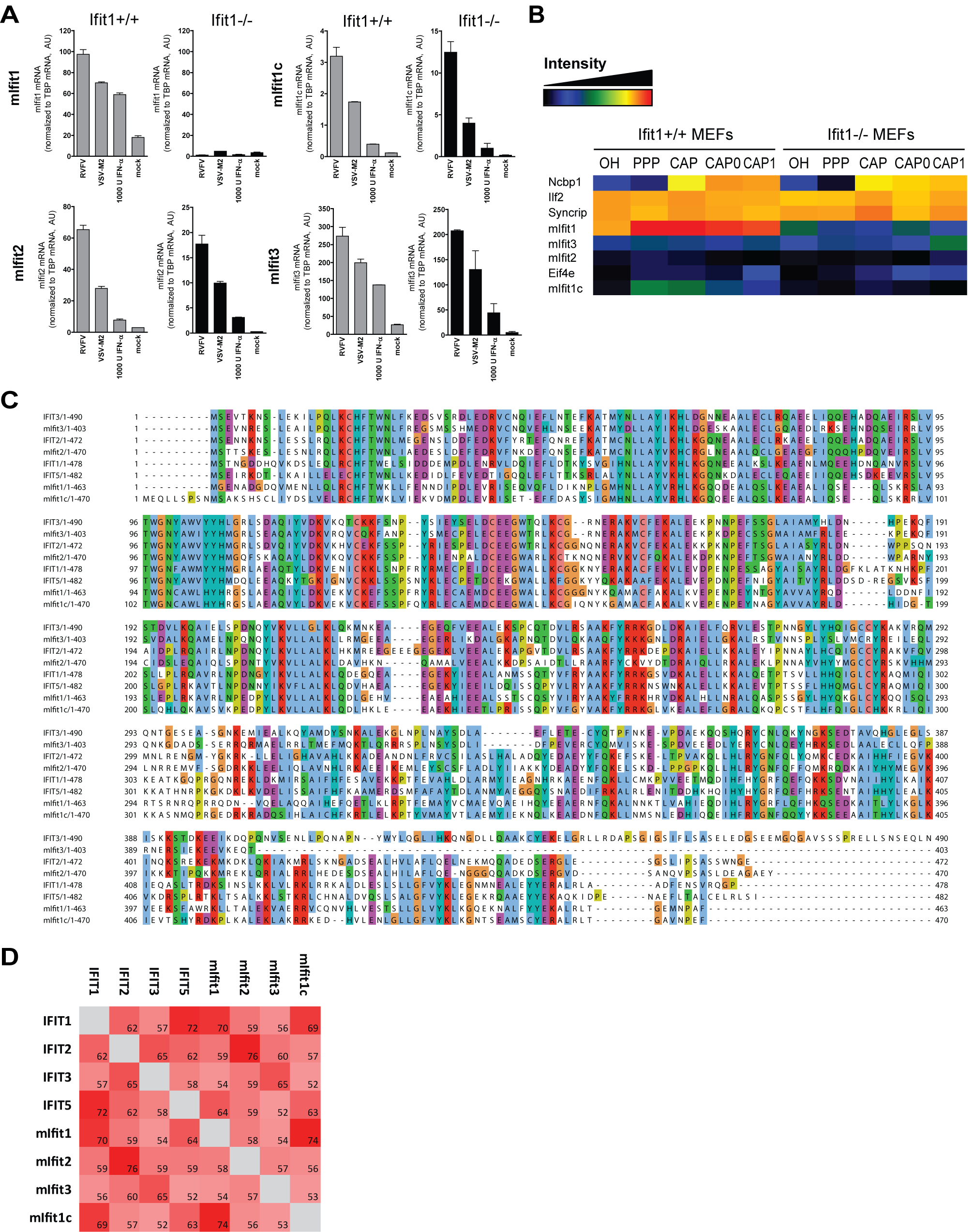

Supplement: Figure S4 — Characterisation of the murine IFIT complex. (a) Expression of Ifit genes in wild-type (Ifit1+/+) and Ifit1-deficient (Ifit1−/−) mouse embryonic fibroblasts (MEFs). MEFs were left untreated, treated with 1000 U/ml IFN-α, or infected with Rift Valley fever virus Clone13 or a mutant version of vesicular stomatitis virus (VSV-M2) at a multiplicity of infection of 1 or 0.01, respectively. Sixteen hours later RNA was analysed by quantitative RT-PCR for mIfit1, mIfit1c, mIfit2 and mIfit3. In each case, one representative experiment of three is shown, with means ±SD after normalization to the TATA-binding protein (TBP) mRNA. (b) Heatmap of selected proteins identified in RNA affinity purifications from cell lysates of Ifit1+/+ and Ifit1−/− MEFs. The plot shows the means of log-transformed label-free quantitation protein intensities in rainbow colours (see colour scale). (c) Alignment of murine and human IFIT proteins using ClustalW. (d) Matrix showing amino acid similarity (based on ClustalW alignment) of all murine and human IFIT proteins. Percent similarity is indicated as color coded from white to red, and the exact similarity is shown within each element of the matrix. (TIF) [file ppat.1003663.s004.tif]

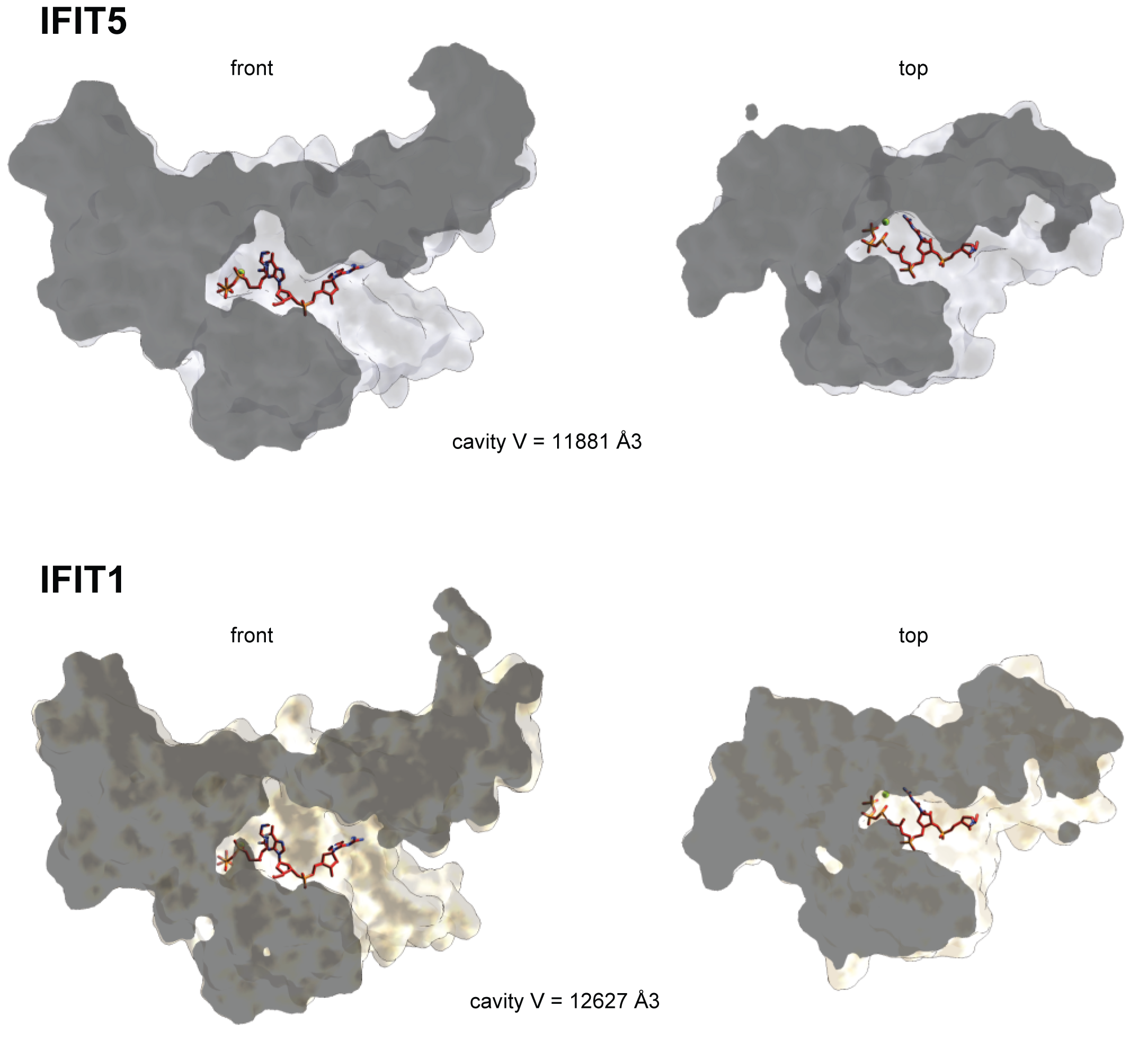

Supplement: Figure S5 — Comparison of the RNA binding cavities of IFIT5 and IFIT1. Sections of surface representations of the solvent-accessible surfaces of IFIT5 (top) and IFIT1 (bottom) are shown, with PPP-RNA bound as in IFIT5 (stick representation, superimposed on IFIT1), and the corresponding cavity volumes V calculated as described in Materials and Methods. In our calcuations, the main RNA-binding cavity in IFIT5 has volume of 11881 Å3. The calculated volume of the corresponding cavity of the modelled IFIT1, at 12627 Å3, is about 700 Å3 larger. (TIF) [file ppat.1003663.s005.tif]

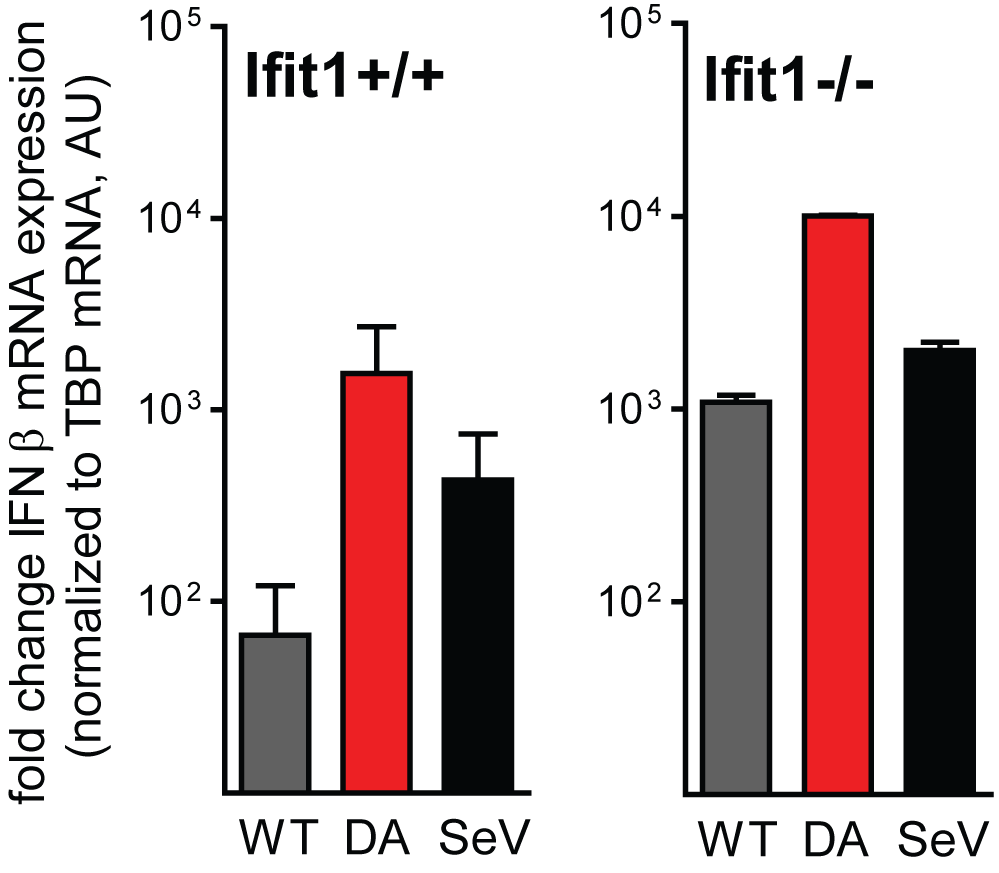

Supplement: Figure S6 — Induction of interferon-β in wild-type and Ifit1-deficient mouse cells. Interferon-stimulated bone marrow-derived macrophages (MΦs) from C57/BL6 (Ifit1+/+) or Ifit1-deficient (Ifit1−/−) mice were left untreated, or infected with wild-type MHV (WT), 2′O-methyltransferase-deficient MHV (DA), or Sendai virus (SeV). Twelve hours later total RNA was harvested and analysed by quantitative RT-PCR for interferon β (IFN-β) mRNA. Data from three independent experiments showing fold change relative to untreated cells (mean ±SD) after normalization to the TATA-binding protein (TBP) mRNA. (TIF) [file ppat.1003663.s006.tif]

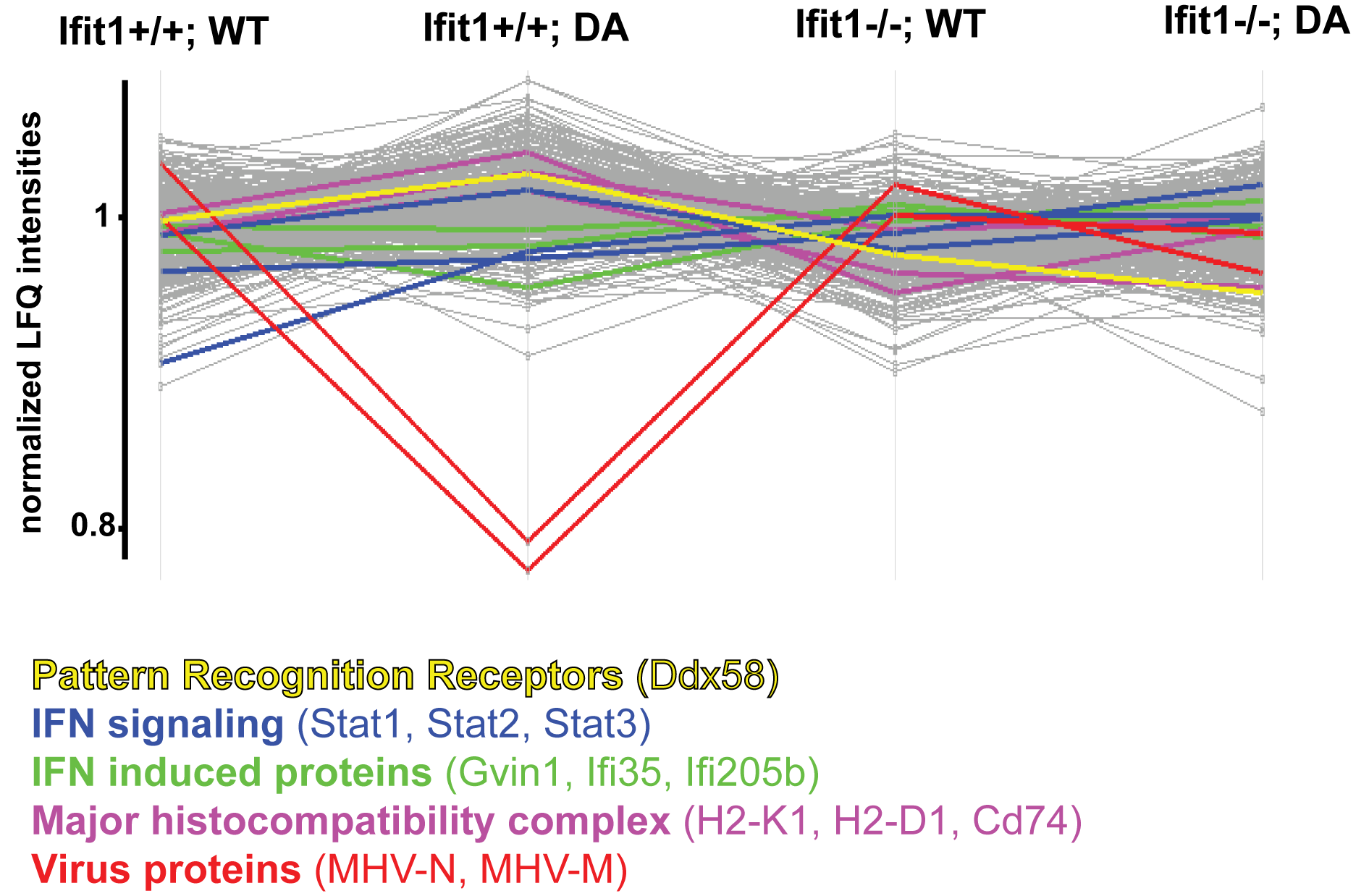

Supplement: Figure S7 — Translation profiles of individual proteins in MHV-infected macrophages. Translation profiles based on pulsed SILAC of macrophages from C75/BL6 (Ifit1+/+) and Ifit1-deficient (Ifit1−/−) mice infected with wild-type MHV (WT) or 2′O methyltransferase-deficient MHV (DA) as shown in Fig. 5. The profile plot shows normalized LFQ intensities of heavy proteins, representing a total number of 451 proteins labelled during the 2 h pulse period. Data show average LFQ intensities from three independent replicates. Selected profiles are coloured and represent MHV proteins and cellular proteins involved in immune responses. (TIF) [file ppat.1003663.s007.tif]
